# Carbon source-induced reprogramming of the cell wall proteome and secretome modulates the adherence and drug resistance of the fungal pathogen *Candida albicans*

**DOI:** 10.1002/pmic.201200228

**Published:** 2012-10-29

**Authors:** Iuliana V Ene, Clemens J Heilmann, Alice G Sorgo, Louise A Walker, Chris G de Koster, Carol A Munro, Frans M Klis, Alistair J P Brown

**Affiliations:** 1 Aberdeen Fungal Group, School of Medical Sciences, Institute of Medical Sciences, University of AberdeenAberdeen, United Kingdom; 2 Swammerdam Institute for Life Sciences, University of AmsterdamAmsterdam, The Netherlands

**Keywords:** Antifungals, Cell wall proteome, *Candida albicans*, Secretome, Stress resistance, Microbiology

## Abstract

The major fungal pathogen *Candida albicans* can occupy diverse microenvironments in its human host. During colonization of the gastrointestinal or urogenital tracts, mucosal surfaces, bloodstream, and internal organs, *C. albicans* thrives in niches that differ with respect to available nutrients and local environmental stresses. Although most studies are performed on glucose-grown cells, changes in carbon source dramatically affect cell wall architecture, stress responses, and drug resistance. We show that growth on the physiologically relevant carboxylic acid, lactate, has a significant impact on the *C. albicans* cell wall proteome and secretome. The regulation of cell wall structural proteins (e.g. Cht1, Phr1, Phr2, Pir1) correlated with extensive cell wall remodeling in lactate-grown cells and with their increased resistance to stresses and antifungal drugs, compared with glucose-grown cells. Moreover, changes in other proteins (e.g. Als2, Gca1, Phr1, Sap9) correlated with the increased adherence and biofilm formation of lactate-grown cells. We identified mating and pheromone-regulated proteins that were exclusive to lactate-grown cells (e.g. Op4, Pga31, Pry1, Scw4, Yps7) as well as mucosa-specific and other niche-specific factors such as Lip4, Pga4, Plb5, and Sap7. The analysis of the corresponding null mutants confirmed that many of these proteins contribute to *C. albicans* adherence, stress, and antifungal drug resistance. Therefore, the cell wall proteome and secretome display considerable plasticity in response to carbon source. This plasticity influences important fitness and virulence attributes known to modulate the behavior of *C. albicans* in different host microenvironments during infection.

## 1 Introduction

The polymorphic fungus *Candida albicans* is a major pathogen of humans. It grows as a commensal organism in the oral cavity, gastrointestinal (GI) and urogenital tracts of most individuals [1] but when the immune system is weakened this fungus can become pathogenic, invade host tissue and cause disease. In susceptible individuals, *C. albicans* causes a wide range of infections, from mucosal infections such as vaginitis and oral thrush, to life-threatening systemic infections [2].

Effective environmental adaptation is an essential feature of medically important pathogens, allowing them to thrive in diverse environments within their mammalian host. *C. albicans* can occupy a variety of niches in humans, many of which contain a range of different carbon sources. Metabolic and stress adaptation represent vital fitness attributes that have evolved alongside virulence attributes in *C. albicans*. Indeed, there is clear evidence for the coordinated regulation of fitness and virulence genes in a niche-specific fashion [3–10].

Recent work has highlighted the importance of carbon metabolism for fungal pathogenicity [11–13]. During the colonization of the GI tract, saccharides such as glucose or fructose are sometimes available to *C. albicans* [14]. However, some host niches, such as mucosal or skin surfaces, contain low concentrations of sugars, and therefore other non-fermentable carbon sources become essential for growth and metabolism of the fungus [5,9]. These include amino acids, fatty acids, and carboxylic acids such as lactic acid. Lactic acid is present in ingested foods, generated by lactic acid bacteria in the GI and urogenital tracts [15], and produced via host metabolic activity [16]. For *Candida glabrata*, lactate assimilation is necessary for proliferation in the intestinal tract and this carboxylic acid is the preferred carbon source in such hypoxic conditions [17]. Following phagocytosis by macrophages, *C. albicans* cells induce alternative pathways of carbon metabolism, such as gluconeogenesis, the glyoxylate cycle, and fatty acid β-oxidation [5,9,18,19]. During systemic infections, *C. albicans* has access to the glucose present in the blood stream, but this sugar is absent in the microenvironments that are invaded during organ infections. A significant proportion of fungal cells infecting the kidney express both glycolytic and gluconeogenic functions [9] and inactivation of the glyoxylate cycle attenuates *C. albicans* virulence during murine systemic candidiasis [5]. Despite the fundamental importance of carbon source to growth and pathogenicity, the impact of alternative carbon sources upon the stress resistance and virulence of *C. albicans* is largely unexplored.

The *C. albicans* cell wall is the first point of contact with the host and a vital protective shield for the fungus, representing a critical mechanistic link between fungal stress resistance and virulence [20]. The cell wall is constructed from chitin, glucan, and mannoproteins. Cell wall mannoproteins, together with those secreted into the external milieu, promote host adhesion, tissue invasion, nutrient uptake, biofilm formation, and modulate immune responses [21–23]. The early steps of infection involve adherence of *C. albicans* to host cells, a process largely mediated by cell surface adhesins that include the agglutinin-like sequence (ALS), hyphal wall, and hyphal-specific regulation protein families [24–26]. Secreted proteins, such as lipases and proteases, facilitate invasion by degrading host tissue and mediating nutrient uptake [23].

When *C. albicans* cells grow in serum or blood, their cell wall architecture is altered, in part by modulating mannosylation patterns [27]. The *C. albicans* cell wall undergoes dramatic remodeling in response to both serum and carbon source [28]. Growth on lactate affects the architecture of the glucan and mannan layers of the cell wall [28] and this remodeling correlates with significant differences in adaptation and resistance to osmotic stress, cell wall stresses, and antifungal drugs.

We predicted that this major cell wall remodeling in response to carbon source extends to the cell wall proteome and secretome because the *C. albicans* cell wall proteome is known to be dynamic. In particular, the complement of glycosylphosphatidylinositol (GPI)-anchored proteins has been shown to respond to ambient pH [22,29,30]. Furthermore, those *C. albicans* proteins that are released into the milieu (the secretome) vary extensively in response to growth conditions, cell wall stress, and antifungal treatment [31,32]. These changes are thought to affect the virulence of *C. albicans* by modulating adherence, invasion and nutrient uptake [21–23]. Therefore we also predicted that the effects of alternative carbon sources upon the stress resistance and antifungal drug sensitivity of *C. albicans* would be mediated in part by changes in the cell wall proteome and secretome.

We tested our predictions by examining the impact of carbon source on the *C. albicans* cell wall proteome and secretome using a proteomic approach. Cell wall and secreted proteins whose levels differ significantly between lactate- and glucose-grown cells were identified. The influence of these target proteins on the resistance of *C. albicans* to cell wall stresses, osmotic stress, and antifungal drugs was tested. We have also examined whether these proteins influence adherence and biofilm formation in a carbon source-dependent fashion. Significantly, we show that many of the cell surface proteins that are regulated by carbon source contribute to stress resistance, adherence, and biofilm formation, suggesting that the differential availability of carbon sources in host niches has a significant influence upon the fitness of *C. albicans* cells during disease progression.

## 2 Materials and methods

### 2.1 Strains and growth conditions

Strains used in this study (Supporting Information Table S4) were grown at 30°C in minimal medium containing 2% carbon source (glucose, lactate, or glucose plus lactate each at 1%), 0.67% yeast nitrogen base without amino acids, and supplemented with 10 μg/mL of the appropriate auxotrophic requirements. Growth curves for these conditions have been published previously [28]. Cells were grown overnight at 30°C, 200 rpm, diluted to an OD_600_ of 0.1 in fresh medium, and harvested at midexponential phase (OD_600_ = 0.4–0.5) for analyses and sensitivity assays. The final pH of the cultures was 5.2–5.6. Where necessary, the CIp10 (*CaURA3*) vector was integrated at the *RPS1* locus in mutants to generate *URA3* derivatives [33,34].

### 2.2 Freeze substitution transmission electron microscopy

Protocols for freeze substitution transmission electron microscopy were performed as described [35] except that *C. albicans* cells were harvested by filtration rather than centrifugation to maximize cell wall integrity, and ultrathin sections were cut at a thickness of 100 nm. Samples were imaged with a Philips CM10 transmission microscope (FEI UK Ltd.) equipped with a Gatan 600 W camera and images were recorded using Digital Micrograph (Gatan Inc., Abingdon Oxon, UK). At least 30 cells were visualized for each sample.

### 2.3 Cell wall and secretome preparation

*Candida albicans* RM1000 cells grown to midexponential phase were harvested by centrifugation. The resulting supernatant was sterile filtered and then concentrated on 10-kD cut-off filters (Amicon Ultra-15 Centrifugal filter units, Millipore) as described previously [31]. To analyze the wall proteome, cell walls were isolated by hot SDS extraction. The cell pellet obtained was used to prepare cell walls as described previously [36]. Briefly, the finely ground cell pellet was washed several times with PBS, and then subjected to breakage in a FastPrep bead beater (Savant Instruments Inc., Farmingdale, NY, USA) with glass beads (0.25–0.50 mm, 12–16 runs for 45 s at speed 6) in the presence of a protease inhibitor cocktail. Full breakage was controlled by light-microscopic inspection. The pellet was washed several times with 1 M NaCl and stored overnight at 4°C. The following day, the pellet was washed several times with MilliQ-water and then boiled four times for 10 min in SDS extraction buffer (150 mM NaCl, 2% w/v SDS, 100 mM Na-ethylenediaminetetraacetic acid, 100 mM β-mercaptoethanol, 50 mM Tris-HCl, pH 7.8), washed with MilliQ-water and lyophilized overnight. The resulting purified wall pellets were either stored at −80°C or directly reduced and *S*-alkylated [37].

### 2.4 Mass spectrometric analyses of cell wall proteomes and secretomes

This was carried out essentially as described in [38]. Lyophilized cell walls were reduced with 10 mM DTT in 100 mM NH_4_HCO_3_ (1 h at 55°C). After cooling to room temperature and centrifugation, the supernatant was discarded. The reduced proteins were alkylated with 65 mM iodoacetamide in 100 mM NH_4_HCO_3_ for 45 min at room temperature in the dark. The samples were quenched with 55 mM DTT in 100 mM NH_4_HCO_3_ for 5 min. Subsequently, the samples were washed six times with 50 mM NH_4_HCO_3_ and either frozen in liquid nitrogen and stored at −80°C or digested using 2 μg Trypsin Gold (Promega, Madison, WI) from a 1 μg/μL stock solution for 18 h at 37°C. The concentrated secretome samples were treated similarly, with reduction and alkylation on 10-kD cut-off spin filters (Amicon, Billerica, MA) [32].

The resulting tryptic digests were desalted using a C18 tip column (Varian, Palo Alto, CA) according to the manufacturer's instructions. After evaporation of acetonitrile with a Speedvac (Genevac, Ipswich, England) the peptide concentration was determined at 205 nm using a NanoDrop ND-1000 (Isogen Life science, IJsselstein, The Netherlands) [39]. Each sample was diluted with 0.1% trifluoroacetic acid to a final concentration of 25 ng/μL, and 10 μL per run were injected onto an Ultimate 2000 nano-HPLC system (LC Packings, Amsterdam, The Netherlands) equipped with a PepMap100 C18 reversed phase column (75 μm id, 25 cm length; Dionex, Sunnyvale, CA). An elution flow rate of 0.3 μL/min was used along a linear gradient with increasing acetonitrile concentration over 45 min. The eluting peptides were directly ionized by electrospray in a Q-TOF (Micromass, Whyttenshawe, UK). Survey scans were acquired from *m/z* 350–1200. For low energy collision-induced dissociation (MS/MS), the most intense ions were selected in a data-dependent mode.

### 2.5 Data processing and analysis

After processing with the MaxEnt3 algorithm (MasslynxProteinlynx), the spectra were converted into pkl (peak list) files. Proteins were identified using MASCOT server 2.3.02 (Matrix Science, UK) and a database (6210 entries) consisting of a complete ORF translation of *C. albicans* and a list of regularly encountered contaminants. Enzyme was set to trypsin and two miscleavages were allowed. Carbamidomethyl (C) was used as a fixed modification and Oxidation (M) as a variable modification. Mass tolerance and MS/MS tolerance were set to 0.5 Da. Detected masses were charge deconvoluted to +1. Based on probabilistic MASCOT scoring a *p* value of less than 0.05 was considered significant for peptide identification. The average false discovery rate against a scrambled decoy database was 0.78%. At least three independently obtained biological samples were analyzed for each condition and each was subjected to two MS/MS runs. The MASCOT output (.dat) files were converted per condition to PRIDExml using the PRIDE converter 2.5.5 and uploaded to PRIDE (http://www.ebi.ac.uk/pride/ accession numbers 27058–27063). All identified proteins were subjected to signal peptide prediction using SignalP3.0 [40] and prediction of a GPI-anchor sequence using the BIG-PI fungal predictor [41]. For a semiquantitative analysis, the number of peptides detected for each identified protein was divided by the total number of identified peptides in the respective biological replicate and multiplied by 100 to obtain the % spectral count. Spectral counts for the three growth conditions were compared using two tailed, two sample Student's *t*-test with a significance cut-off of 0.05. These % spectral counts were averaged between the different biological replicates and used to calculate fold changes between the three conditions (glucose, lactate, and glucose plus lactate). For each growth condition, the minimum level of detection was one peptide. The median CV for the wall proteome was 10.1% and for the larger secretome 13.25% (Supporting Information Tables S5 and S6). In order to generate fold ratios between conditions for proteins exclusive to one growth condition, the minimum % spectral count (corresponding to one identified peptide) was used as a substitute for the growth condition in which that protein was below detection levels. Consequently, the final fold change received the “less than” (<) or “more than” (>) symbols. Fold changes between lactate and glucose samples were the basis for selection of protein target for further phenotypic analysis using the following cut-offs: >1.5 for upregulated proteins and <0.66 for downregulated proteins. In addition, a set of proteins that were exclusive to a specific growth condition were also included in the mutant analysis.

### 2.6 Stress phenotypes

Antifungal drug susceptibility was analyzed by treating midexponential *C. albicans* cells grown on the specified carbon source with tunicamycin (4 μg/mL), caspofungin (0.08 μg/mL), amphotericin B (Ambisome 10 μg/mL) or miconazole (25 μg/mL) for 1 h at 30°C, 200 rpm. Cells were then serially diluted and plated onto yeast peptone dextrose agar. CFUs were quantified and antifungal drug sensitivities were calculated relative to those observed for the appropriate parental strains grown under the same conditions. To examine hyperosmotic stress, midexponential *C. albicans* cells were exposed to 2 M NaCl for 1 h, at 30°C, 200 rpm, and then CFUs measured relative to untreated control cells. Means ± SEM for at least three independent experiments were calculated.

To examine resistance to cell wall stresses, serial dilutions of midexponential *C. albicans* cells were plated onto agar from 5 × 10^7^ cells per spot and diluted 1/10 thereafter. The yeast nitrogen base agar contained the specified carbon source, appropriate auxotrophic requirements and was supplemented with Congo Red (CR) (300 μg/mL) or calcofluor white (CFW) (200 μg/mL). The plates were examined after 2–5 days incubation at 30°C and scored based on the number of dilutions growing on the respective plate (out of six dilutions). These scores were averaged for three independent experiments, each with technical duplicates and were used for comparison with the corresponding parental strains.

For antifungal drug, stress, and osmotic stress resistance, each mutant analyzed was compared against its corresponding parental strain and significant changes were determined using *t*-tests (*p* < 0.05) and a minimum of 0.8-fold decrease or 2.5-fold increase in resistance relative to the parental strain. Phenotypes that met these criteria were marked in color (Tables 1 and 2) according to the severity of the phenotype. Values represent the fold change in the resistance of the mutant strain relative to its parental under the same stress conditions.

**Table 1:** Functional analysis of proteins significantly induced or repressed by nonfermentative growth—antifungal sensitivity. Functional analysis (antifungal drug susceptibility) of proteins induced or repressed in the cell wall proteomes or secretomes of lactate- and glucose-grown *C. albicans* cells

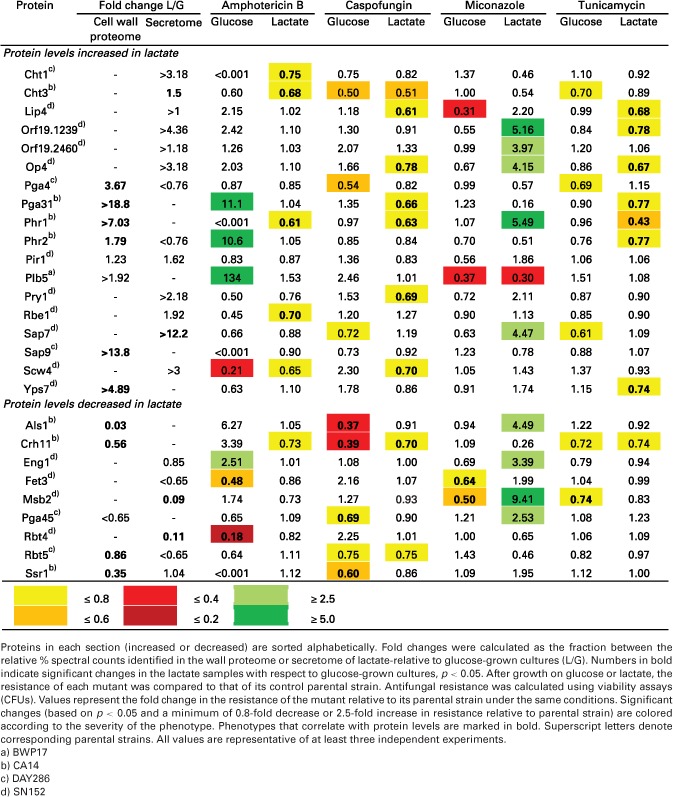

Proteins in each section (increased or decreased) are sorted alphabetically. Fold changes were calculated as the fraction between the relative % spectral counts identified in the wall proteome or secretome of lactate-relative to glucose-grown cultures (L/G). Numbers in bold indicate significant changes in the lactate samples with respect to glucose-grown cultures, *p* < 0.05. After growth on glucose or lactate, the resistance of each mutant was compared to that of its control parental strain. Antifungal resistance was calculated using viability assays (CFUs). Values represent the fold change in the resistance of the mutant relative to its parental strain under the same conditions. Significant changes (based on *p* < 0.05 and a minimum of 0.8-fold decrease or 2.5-fold increase in resistance relative to parental strain) are colored according to the severity of the phenotype. Phenotypes that correlate with protein levels are marked in bold. Superscript letters denote corresponding parental strains. All values are representative of at least three independent experiments.

a) BWP17

b) CA14

c) DAY286

d) SN152

**Table 2:** Functional analysis of proteins significantly induced or repressed by non-fermentative growth—stress resistance and adherence to a plastic surface. Functional analysis (stress resistance and adherence to plastic surfaces) of proteins induced or repressed in the cell wall proteomes or secretomes of lactate- and glucose-grown *C. albicans* cells

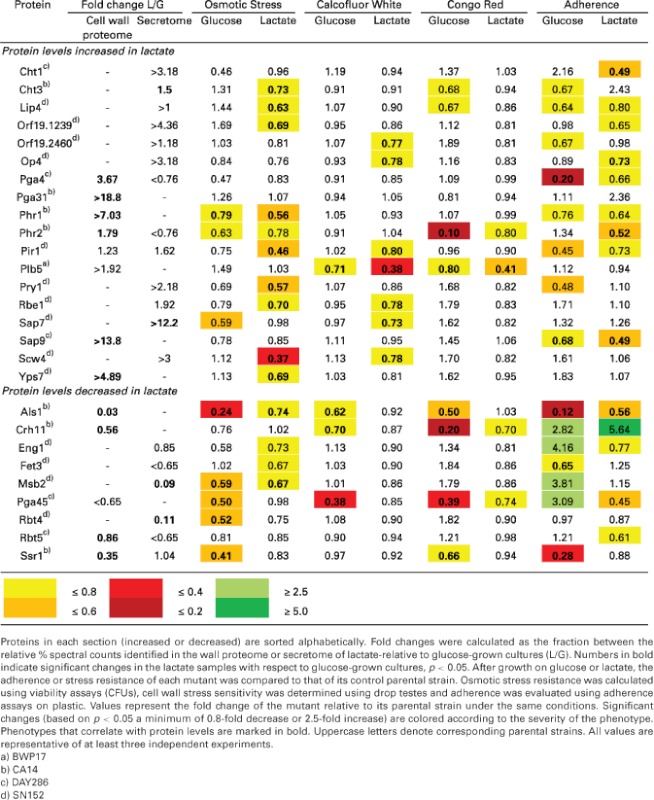

Proteins in each section (increased or decreased) are sorted alphabetically. Fold changes were calculated as the fraction between the relative % spectral counts identified in the wall proteome or secretome of lactate-relative to glucose-grown cultures (L/G). Numbers in bold indicate significant changes in the lactate samples with respect to glucose-grown cultures, *p* < 0.05. After growth on glucose or lactate, the adherence or stress resistance of each mutant was compared to that of its control parental strain. Osmotic stress resistance was calculated using viability assays (CFUs), cell wall stress sensitivity was determined using drop testes and adherence was evaluated using adherence assays on plastic. Values represent the fold change of the mutant relative to its parental strain under the same conditions. Significant changes (based on *p* < 0.05 a minimum of 0.8-fold decrease or 2.5-fold increase) are colored according to the severity of the phenotype. Phenotypes that correlate with protein levels are marked in bold. Uppercase letters denote corresponding parental strains. All values are representative of at least three independent experiments.

a) BWP17

b) CA14

c) DAY286

d) SN152

### 2.7 Cell adhesion

Midexponential *C. albicans* cells were washed twice with dH_2_O and resuspended in PBS. A total of 10^7^ cells/mL in 2 mL PBS were added to 12-well plates (nontreated polystyrene; Costar, Corning Inc., Ewloe, Flintshire, UK) and allowed to adhere for 1 h at 37°C without shaking. After washing three times with PBS, adhered cells were scraped off the plastic surface into 1 mL PBS and quantified by OD_600_ and counting colony forming units (CFUs). OD_600_ and CFUs displayed the same trends. Results presented in Fig. 3A and Table 2 represent the average CFUs ± SEM for at least three independent experiments for each growth condition, each with technical duplicates.

**Figure:**
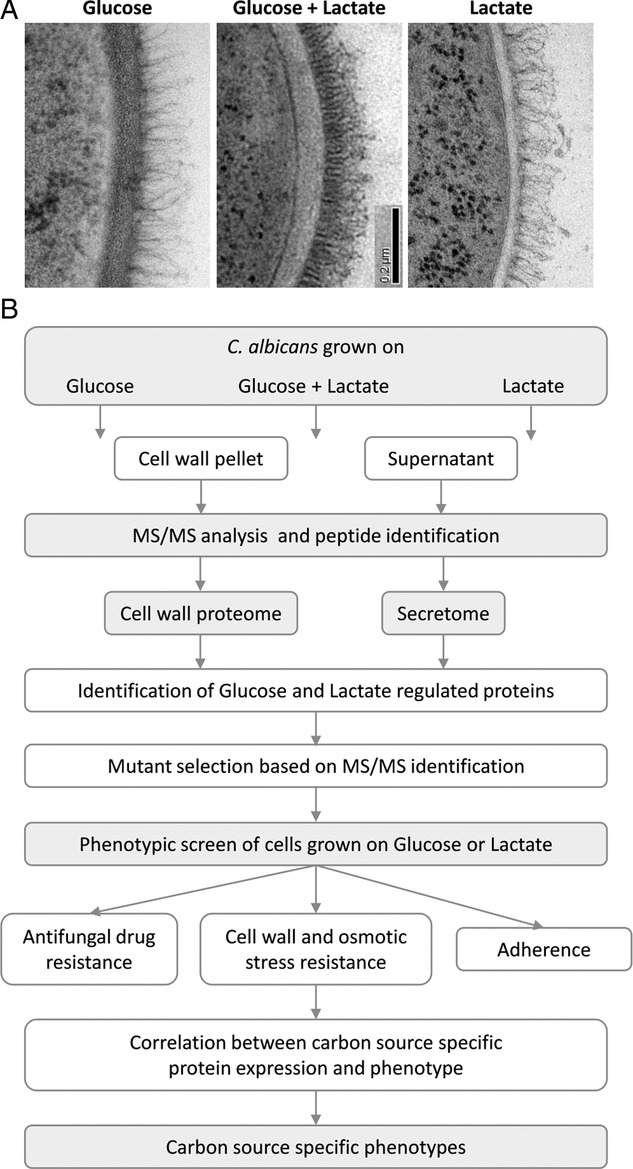
(A) Transmission electron microscopy of *C. albicans* cell walls after growth on glucose, lactate, or a mix of glucose plus lactate (scale bar, 0.2 μm, *n* = 50) reveal the impact of carbon source on cell wall architecture. (B) Schematic of the experimental design used to examine the cell wall proteome, secretome, and carbon source-specific phenotypes.

**Figure:**
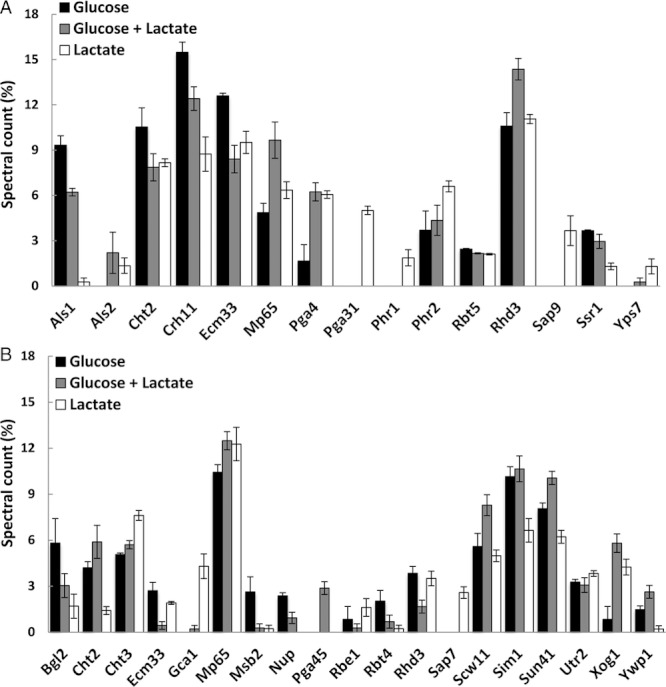
Significant changes in the wall proteome (A) and secretome (B) of *C. albicans* cells induced by growth on glucose, lactate, or glucose plus lactate. Differences in protein levels between the three conditions (*p* < 0.05) were calculated using the averaged % spectral counts from three to four independent biological replicates, each with two technical runs.

**Figure:**
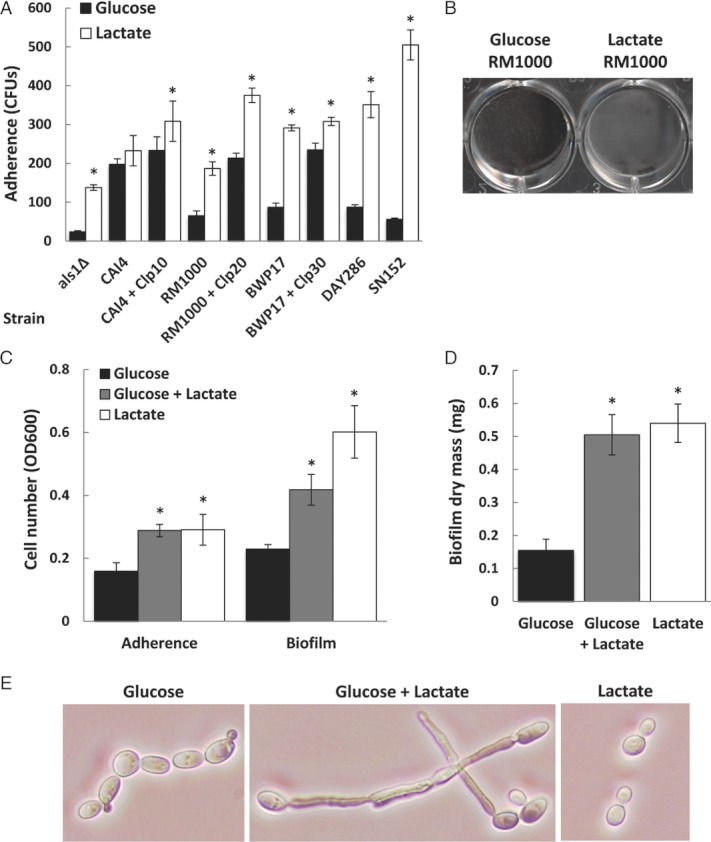
Carbon source affects *C. albicans* adherence and biofilm formation. (A) Adherence of wild type *C. albicans* strains grown on either glucose or lactate was assayed by measuring the number of cells (OD_600_ and CFUs) adhered to a plastic surface after 1 h static incubation at 37°C. Bars represent averaged CFUs ± SEM of at least three independent experiments. (B) *C. albicans* RM1000 cells grown in glucose or lactate display different levels of adherence to a plastic surface after 1 h. (C) Number of cells (OD_600_) adhered to a plastic surface (1 h, 37°C) or in biofilms (24 h, 37°C). Results represent the means ± SEM of four to six experiments, *p < 0.05 relative to glucose-grown controls. (D) Dry mass of biofilms formed on silicone in minimal medium (24 h, 37°C). Results represent the means ± SEM of ten experiments, *p < 0.05 relative to glucose-grown biofilms. (E) Morphology of cells recovered from biofilms on plastic surface grown on glucose, lactate, or glucose plus lactate (24 h, 37°C).

The same adherence assay was used for phenotypic analysis of target proteins. Each mutant analyzed was compared against its corresponding *URA3* parental strain and significant changes were determined using *t*-tests (*p* < 0.05) and a minimum 0.8-fold decrease or 2.5-fold increase in adherence relative to the parental strain. Phenotypes that met these criteria were marked in color (Table 2) according to the severity of the phenotype. Values represent the fold change in adherence of the mutant strain relative to its parental under the same stress conditions.

### 2.8 Biofilm assays

Biofilm assays on silicone were adapted from Nobile et al. [42,43]. The silicone squares (1.5 × 1.5 cm) were weighed beforehand, incubated overnight at 37°C and 150 rpm agitation with bovine serum albumin (5%, in PBS) and washed with PBS. *C. albicans* RM1000 cells were grown overnight at 37°C in yeast nitrogen base minimal medium containing the appropriate auxotrophic requirements (10 μg/mL) and 2% carbon source. The cultures were diluted to an OD_600_ of 0.1 in the morning and grown to midexponential phase. The cells were harvested, diluted to an OD_600_ of 0.5 in 2 mL medium and added to a sterile 12-well plate with prepared silicone squares. The inoculated plates were incubated for 90 min at 37°C and 150 rpm agitation for initial adhesion of cells. To remove nonadherent cells, the squares were washed with 2 mL of PBS and incubated for an additional 24 h in the same conditions. After 24 h the medium was removed and the squares were dried overnight in a fume hood. The final mass of the squares was determined in order to assess the efficiency of cell adherence and ability to form a biofilm (biofilm dry mass). Results represent the average biomass ± SEM from ten independent experiments, each performed with technical duplicates.

Assays of biofilm formation on plastic were adapted from a similar method [44]. *C. albicans* RM1000 cells were grown to midexponential phase in minimal medium. A total of 10^7^ cells/mL were inoculated in 2 mL of minimal medium into wells of 12-well plates. After 24 h incubation at 37°C with no shaking, the bottoms of the dishes were gently rinsed twice with PBS to remove nonadhering cells. Adherent cells were scraped off with a toothpick and the number of cells determined by measuring OD_600_. Cells were examined microscopically and photographed. The average number of cells in the biofilm ± SEM was determined from a total of four independent experiments, each performed with technical duplicates.

### 2.9 Microscopy

The morphology of cells recovered from biofilms on plastic (after 24 h static incubation at 37°C) was examined by differential interference contrast microscopy microscopy with a Zeiss Axioskop microscope. Images were recorded using a JVC KY-F1030 digital camera. At least 200 cells were examined from four independent biological replicates.

### 2.10 Western blotting

Protein extracts were prepared from midexponential *C. albicans* cells grown on either glucose or lactate and subjected to Western blotting as described before [45]. Mkc1 and Cek1 activation were detected using a phospho-specific phospho-p44/42 MAPK (Erk/12; Thr202/Tyr204) antibody 4370 (New England Biolabs). The secondary antibody was HRP-labeled anti-rabbit immunoglobulin G 7074 (New England Biolabs), which was detected using Pierce enhanced chemiluminescence PlusTM Western blotting reagents (Thermo Scientific, Cramlington, UK). The two experiments presented are representative of six biological replicates.

### 2.11 Statistical analyses

Results from independent replicate experiments are expressed as means ± SEM. Statistical significance was calculated using Excel *t*-test and a significant difference was noted for *p* < 0.05.

## 3 Results and discussion

### 3.1 Impact of carbon source on the *C. albicans* proteome and secretome

We have shown previously that the growth of *C. albicans* yeast cells on glucose or on lactate as the sole carbon source has a major impact upon their cell wall architecture (Fig. 1A) [28]. Given the dynamic nature of the fungal cell wall proteome [20], we predicted that this change in carbon source would exert corresponding changes in the wall proteome and secretome, and that these changes underlie many of the cell wall phenotypes described for glucose- and lactate-grown cells [28]. Therefore, we performed mass spectrometric (LC-MS/MS) analyses of the secretome and cell wall proteome of *C. albicans* cells grown to midexponential phase in minimal medium containing glucose, lactate, or a mix of these two carbon sources (Fig. 1B). Peptide identifications were performed in a minimum of three biological replicates each with two technical replicates. Proteins were identified matching the obtained MS/MS data to the complete ORF translation of *C. albicans*. For proteins from which only a single peptide had been identified the spectra were manually inspected and only included when this peptide was identified in at least two independent biological replicates. To correct for loading differences and to estimate relative protein abundances, peptide identifications from each biological replicate and for each protein were divided by the total number of identified peptides [46,47]. The obtained percent peptide identifications (% spectral count) were averaged per growth condition and used for comparing the different carbon sources. The datasets can be accessed through the PRIDE database (http://www.ebi.ac.uk/pride: 27058–27063).

All secretomes and wall proteomes were highly enriched for proteins containing signal peptides. Less than 2% of all peptides identified in the secretome and cell wall proteome were of proteins without secretion signal, and the majority of wall proteins consisted of GPI proteins. These observations are consistent with previous studies [31,32,36]. Lactate- and glucose-grown *C. albicans* cells displayed major differences in their profiles of secreted and cell wall proteins (Fig. 2, Supporting Information Tables S1 and S2). Many of these differences indicated that the change in carbon source affected specific cellular processes associated with the cell surface, such as cell wall remodeling, adherence, biofilm formation, cell type, and metabolism. Differential protein expression in these functional categories is described below.

#### 3.1.1 Cell wall remodeling

Proteins with functions associated with cell wall remodeling were expressed at higher levels in the cell wall proteomes and secretomes of *C. albicans* cells grown on lactate (Supporting Information Table S3). This was consistent with higher basal levels of Mkc1 activation in lactate- compared to glucose-grown cells (Supporting Information Table S3). This was consistent with higher basal levels of Mkc1 activation in lactate- compared to glucosegrown cells (Supporting Information Fig. S1). Lactate-grown cells displayed increased levels of the chitinases Cht1 and Cht3 [48–50] (Supporting Information Table S3). In contrast, Cht2 was found at higher levels in the cell wall proteomes and secretomes of glucose-grown cells (Fig. 2). Chitin is present in similar proportions in the cell walls of glucose and lactate-grown cells [28]. Therefore, we suggest that changes in chitin architecture or chitin-glucan cross-links, rather than alterations in absolute chitin levels, may contribute to the increased stress resistance of the lactate-grown cells.

The proteomes and secretomes of lactate-grown cells also displayed elevated levels of proteins involved in β-glucan remodeling. These remodeling enzymes included glucanosyltransferases (Pga4, Phr1, Phr2), an exo-glucanase (Xog1) and a 1,3-β-glucan-linked structural protein (Pir1) (Table 1). β-1,3-glucan and β-1,6-glucan play cooperative roles in the fungal cell wall, contributing to its rigidity [51]. Therefore the differential β-glucan remodeling enzyme levels of lactate- and glucose-grown cells may affect the relative amounts and linkages of β-glucans in their cell walls, thereby contributing to the major differences in their cell wall architecture and elasticity [28].

Interestingly, both Phr1 and Phr2 were found at higher levels in the cell wall proteome of lactate-grown cells (Fig. 2A). Phr1 and Phr2 process β-1,3-glucans, making acceptor sites available for attachment of β-1,6-glucans [52], and probably act as β-1,3-glucan elongases [53], thus contributing to the remodeling of the β-glucan layer. Although the transcription of *PHR1* and *PHR2* is pH regulated, both genes can be induced as part of a compensatory response to cell wall stress [32], possibly via Mkc1 signaling. Pga31 levels are also elevated in the cell wall proteome of lactate-gown cultures (Fig. 3A). This reinforces the idea that Mkc1 signaling is active under these growth conditions (Supporting Information Fig. S1) because Pga31 is thought to be part of this cell wall salvage pathway [54].

A different set of cell wall remodeling proteins was induced in glucose-grown cells, namely Bgl2 (secreted 1,3-β-glucosyltransferase), Crh11 (cell wall transglycosylase believed to be involved in connecting chitin to β-glucan), Ecm33 (expressed on the cell wall and secreted), Sim1 (secreted), and Ssr1 (β-glucan associated protein with roles in cell wall structure). In contrast, the cell wall damage sensor Msb2 [55] was significantly repressed in the lactate secretome compared with the secretome of glucose-grown cells (Fig. 2B). This was consistent with increased Cek1 activation in the glucose condition (Supporting Information Fig. S1) as Msb2 is required for Cek1 phosphorylation [55].

Because *C. albicans* cells grown on a mixture of glucose plus lactate display relatively high stress and drug resistance, similar to lactate-grown cells [28], we also examined their cell wall proteome and secretome under this growth condition. Many cell wall integrity-related proteins that were upregulated in lactate-grown cells were also observed in this condition (Fig. 2, Supporting Information Tables S1 and S2). In particular, Pga4 and Phr2 were found at elevated levels in the cell wall proteome after growth on lactate or glucose plus lactate (Fig. 2A). Ssr1 and Crh11 were present at relatively high levels in the cell wall proteome (Fig. 2A), and the levels of the chitinases Cht1, Cht2, and Cht3 were elevated in the secretome (Fig. 2B, Supporting Information Table S2). In contrast, other proteins that were induced in the secretome of glucose cultures (Bgl2, Msb2) were present at intermediate or low levels in the mixed medium (Fig. 2B). Overall, our analyses suggest that the complement of cell wall remodeling enzymes expressed during growth on the single carbon sources was partially retained in cells cultivated in the mixed carbon source medium.

#### 3.1.2 Adherence

Lactate-grown cells adhere more strongly to plastic surfaces than glucose-grown cells [28]. Consistent with this observation, members of the ALS family were differentially regulated by carbon source. Als1 was highly induced in the glucose wall proteome (Fig. 2A), whereas Als2 and Als4 were detected at higher levels in the lactate wall proteome and secretome, respectively (Supporting Information Table S3). Als1 contributes to the adherence of *C. albicans* to endothelial cells [56] and its expression is dramatically induced in biofilms [57]. Also, Als1 possibly has redundant functions with Als3 in models of biofilm formation [58]. Als2 is not induced during many in vitro growth conditions [59], but its induction during ketoconazole treatment [60] and cell wall regeneration of protoplasts [61] links this adhesin to cell wall remodeling. Members of the ALS family were also induced at high levels in cells grown on glucose plus lactate, with Als1 and Als2 abundant in their cell wall proteome (Fig. 2A). The elevated levels of these adhesins correlated with the apparent existence of cell–cell contacts between as revealed by transmission electron microscopy (Supporting Information Fig. S2).

Sap9 was also greatly enriched in the lactate cell wall proteome (Fig. 2A). Sap9 is a GPI-anchored member of the secreted aspartyl protease family involved in the proteolytic cleavage and maturation of cell wall and secreted proteins in *C. albicans* [62–64]. Increased Sap9 expression has been associated with adhesion to epithelial cells and epithelial damage during oral infection [62], making it an important mediator of host-pathogen interactions [65].

The Phr1 transglycosylase is involved in cell wall remodeling and in adhesion to abiotic surfaces and epithelial cell monolayers [66]. Therefore the high levels of Phr1 in the lactate wall proteome (Fig. 2A) could also contribute to the increased adhesion of lactate-grown cells to plastic surfaces [28].

Taken together, the differential expression of these adhesins and adhesion-related functions provides potential mechanistic explanations for the effects of carbon source on *C. albicans* adhesion.

#### 3.1.3 Biofilm-related proteins

Almost without exception, any proteins involved in biofilm formation that were detected in our mass spectrometric analyses were enriched in the wall proteome and secretome of lactate- rather than glucose-grown cultures (Supporting Information Table S3). Thus, Gca1, a predicted secreted glucoamylase shown to promote biofilm matrix formation [67], was exclusively detected in the lactate supernatant (Fig. 2B). This was also the case for Sap9 and Scw4 (Supporting Information Table S3). These proteins as well as the ALS family are significantly induced in biofilms [58,68–70]. Based on these observations it is conceivable that lactate-grown cells may be more efficient in forming biofilms than glucose-grown cells. This is not inconsistent with reports that alcohol dehydrogenase (Adh1) contributes to biofilm formation [71] and central carbon metabolism is modulated during biofilm formation [72].

#### 3.1.4 Mating-regulated and opaque-specific proteins

Numerous pheromone-regulated and opaque-specific proteins were identified in the lactate wall proteome and secretome (Supporting Information Table S3). Op4 is thought to be specifically transcribed in opaque-phase cells [73]. More recently Op4 has been detected at low levels in response to antifungals and pH [31,32], and here we observed Op4 in the lactate-grown secretome (Table 1). Therefore, its expression is not restricted to opaque cells but can also be induced in white cells under certain environmental conditions. Pry1 was exclusively identified in the lactate secretome (Supporting Information Table S3). Pry1 is also considered to be opaque specific [74], but its function remains obscure. Pga31, a GPI-linked protein that was significantly enriched in the lactate wall proteome (Fig. 2A), is regulated upon white-opaque switching (C. A. Munro, personal communication) and further induced during cell wall regeneration [61]. We also detected Sap7, Scw4, and Yps7 exclusively in the lactate secretome and cell wall proteome (Supporting Information Table S3). These proteins are regulated in response to pheromone [75,76]. These data are thus consistent with the idea that the regulation of the mating response in *C. albicans* is influenced by environmental cues and that this pathogen integrates metabolic signals with those of the pheromone signaling pathway. Indeed, the responses of *C. albicans* cells to α-pheromone are strongly influenced by nutritional conditions [77].

#### 3.1.5 Metabolism and niche-specific proteins

Proteins with metabolic functions and those involved in niche-specific adaptation were also present at elevated levels during growth on lactate. The secreted lipase, Lip4, was observed exclusively in the lactate secretome (Supporting Information Table S3). *LIP4* expression has been strongly correlated with mucosal infections [78,79]. Sap7 was also exclusive to the lactate secretome (Supporting Information Table S3) and, to our knowledge, no in vitro conditions have been identified that lead to its increased expression. However, *SAP7* is up-regulated during vulvovaginal candidiasis [80] and oral infection [81]. The extracellular glucoamylase, Gca1, was also greatly enriched in the lactate secretome (Fig. 2B). *GCA1* is transcribed during rat oral infection and its transcription is regulated by carbohydrates [67,70]. Moreover, we detected high levels of Pga4 in the wall proteome of lactate-grown cells (Fig. 2A). This transglycosylase is up-regulated in a human epithelium model of oral candidiasis [82] and in mouse liver infections [83].

Gca1, Lip4, and Sap7 were not detectable, or only present at low levels during growth on glucose plus lactate, the mixed medium condition (Supporting Information Table S2). This is consistent with their metabolic roles. In contrast, Pga4 that plays a more general role in wall integrity and remodeling was observed during growth on glucose plus lactate. These observations suggest that the lack of glucose and the reliance on alternative carbon sources contributes significantly to the expression profiles of *C. albicans* cells during mucosal infections.

### 3.2 Impact of carbon source-regulated proteins upon cell surface-related phenotypes

Our data show clearly that changes in carbon source exert significant effects upon the cell wall proteome and secretome of *C. albicans*. The next step was to test whether these changes contribute to phenotypic differences between glucose- and lactate-grown *C. albicans* cells.

We hypothesized that certain proteins in the cell wall or secretome might contribute to the elevated resistance of lactate-grown *C. albicans* cells to cell wall stressors [28]. Furthermore, we predicted that the differential regulation of particular adhesins and biofilm-related proteins might influence adhesion and biofilm formation under these conditions. To test these predictions, we selected target proteins for phenotypic analyses based on our proteomic datasets. We selected cell wall and secreted proteins that displayed significant differences in their expression levels between glucose- and lactate-grown cells (Fig. 1B), choosing targets that represent the functional categories discussed above. *C. albicans* mutants corresponding to each of these protein targets were grown on glucose or lactate as the sole carbon source, and screened for drug and stress sensitivities (Fig. 1B). The expectation was that, if resistance is dependent on a particular protein target, the corresponding mutant should display the appropriate sensitivity in a carbon source-conditional manner. We also examined the impact of carbon source on adherence and biofilm formation.

#### 3.2.1 Susceptibility to antifungal drugs

Lactate-grown cells display increased resistance to amphotericin B, caspofungin, tunicamycin, and increased sensitivity to miconazole [28]. Therefore, we screened for target proteins that contribute to these phenotypes in a carbon source-dependent fashion. The susceptibility of the selected mutants was expressed relative to the corresponding parental strains grown under the same conditions, significant differences being highlighted in color (Table 1). Those conditional phenotypes that correlated with changes in the levels of the corresponding protein were highlighted in bold (Table 1).

Inactivation of Cht1, Cht3, Phr1, and Rbe1 negatively affected the amphotericin B resistance of *C. albicans* cells grown on lactate. In contrast, cells lacking Fet3 or Rbt4 were more sensitive to amphotericin B during growth on glucose (Table 1). This was consistent with the differential regulation of these proteins during growth on lactate or glucose (Supporting Information Table S3). However, *scw4* cells displayed amphotericin B sensitivity irrespective of the carbon source (Table 1) although Scw4 was only detected in the secretome of lactate-grown cells.

The inactivation of Lip4, Op4, Pga31, Phr1, Pry1, or Scw4 increased the sensitivity to caspofungin during growth on lactate, while *als1*, *crh11*, *pga4*, *pga45*, *sap7*, and *ssr1* mutations attenuated caspofungin resistance during growth on glucose (Table 1). In contrast, the inactivation of Cht3 and Rbt5 increased caspofungin susceptibility when cells were grown on either carbon source (Table 1). *CRH11* expression is induced by exposure to caspofungin [60], and Cht3, Crh11, Phr1, Pga4, Pga31, and Ssr1 are regulated during cell wall remodeling 32,52,54,60,84]. Notably, mutation of Op4, Pry1, Sap7, or Scw4, which are reported to be opaque-specific or pheromone-regulated proteins [73–76], also affected caspofungin susceptibility (Table 1).

The inactivation of some target proteins (e.g. Orf19.1239, Phr1, Msb2) decreased the miconazole susceptibility of *C. albicans* cells grown on lactate (Table 1). The basis for this is not clear. The disruption of Fet3, Lip4, and Msb2 significantly attenuated the miconazole susceptibility of glucose-grown cells, while the mutation of Plb5 altered susceptibility irrespective of carbon source (Table 1).

*Candida albicans crh11* cells displayed decreased resistance to tunicamycin on both carbon sources (Table 1), which is consistent with its function as a cell wall transglycosylase [85]. The inactivation of Cht3, Msb2, Pga4, and Sap7 increased the tunicamycin sensitivity of glucose-grown cells (Table 1), whereas the disruption of Lip4, Op4, Orf19.1239, Pga31, Phr1, Phr2, and Yps7 conferred tunicamycin sensitivity during growth on lactate (Table 1). Again, the inactivation of some mating-regulated proteins (Op4, Pga31, Yps7) affected tunicamycin susceptibility.

Taken together, these experiments have revealed carbon source-dependent drug sensitivity phenotypes for many of the *C. albicans* mutants studied. In most cases the carbon source dependence correlated with the expression patterns of the corresponding proteins in the proteomics datasets. This is consistent with the idea that changes in carbon source affect the expression of many cell wall and secreted proteins, significantly impacting upon antifungal drug resistance.

#### 3.2.2 Osmotic stress resistance

The inactivation of several target proteins affected osmotic stress resistance in a carbon source-dependent manner. Cht3, Lip4, Orf19.1239, Phr1, Pir1, Pry1, Rbe1, Scw4, and Yps7, induced during growth on lactate, had a stronger impact on the osmotic stress resistance of lactate-rather than glucose-grown cells (Table 1). Similarly, the disruption of Als1, Msb2, Pga45, Rbt4, or Ssr1 induced in glucose-grown cells, altered the osmotic stress resistance of these cells (Table 1). Therefore, our data are consistent with the working hypothesis that the high osmotic stress resistance of lactate-grown cells [28] is mediated partly by proteins that are induced during growth on lactate.

#### 3.2.3 Cell wall stress phenotypes

To investigate which of the target proteins might play roles in resistance to cell wall stress, we tested the resistance of the *C. albicans* mutants to CFW and CR. These cell wall perturbing agents bind and interfere with the synthesis and cross-linking of chitin and glucan, respectively. The data are summarized in Table 2, and the raw data for mutants displaying consistent defects are presented in Supporting Information Fig. S3.

All of the mutant phenotypes observed during growth on CFW plates correlated with the expression patterns of the corresponding proteins during growth on glucose or lactate (Table 2). As mentioned above, some of these proteins play active roles in cell wall remodeling (Crh11, Pir1), or are stress induced (Pga45, Pir1, Rbe1). Others, such as Op4, Orf19.2460, Sap7, and Scw4, are opaque-specific or mating-regulated proteins.

The inactivation of a different subset of cell wall and secreted proteins affected CR resistance. The inactivation of Als1, Cht3, Crh11, Lip4, Pga45, Phr2, or Ssr1 decreased CR resistance during growth on glucose, while lactate-grown *plb5* cells displayed increased CR sensitivity (Table). Once again, the majority of these mutant phenotypes correlated with the expression patterns of the corresponding proteins in the glucose and lactate cell wall proteomes and secretomes. Given the roles of Cht3, Crh11, Phr2, Pir1, and Ssr1 in cell wall remodeling, these data reinforce the view that carbon source strongly influences the architecture of the cell wall and hence the resistance of *C. albicans* cells to cell wall stress.

#### 3.2.4 Adherence to plastic surfaces

Growth on different carbon sources affects the adhesion of *C. albicans* cells to plastic surfaces [28]. Therefore, we also tested whether any of our target proteins has an impact upon adhesion. All of the parental control strains displayed increased adherence to plastic following growth on lactate rather than glucose (Fig. 3A and B). The adherence of each mutant was expressed relative to the adhesion of its parental control under the equivalent conditions (Table).

Als1 inactivation decreased the adherence of glucose-grown cells to a greater extent than that of lactate-grown cells (Table), suggesting that the relative contribution of Als1 to adherence is carbon source dependent. This was consistent with a previous report indicating that *als1* cells display an adherence defect during growth on glucose [56]. The role of Pga4 in adherence has not been described before, but its function as a β-1,3-glucanosyltransferase and its upregulation in a reconstituted human epithelium model of oral candidiasis [82] indicate its contribution to host-pathogen interactions. Both Phr1 and Sap9 are known to contribute to adherence: *phr1* null mutants display a reduction in adhesion to abiotic surfaces [66], while Sap9 modulates the adhesion to epithelial cells [62].

The inactivation of Cht1, Phr2, and Pga45 strongly decreased the adherence of lactate-grown cells, whereas proteins such as Pry1, and Ssr1 promoted the adherence of glucose-grown cells (Table). Notably, some mating-regulated proteins such as Op4, Orf19.2460, and Pry1 were among those targets that influence *C. albicans* adherence.

In some cases we observed that the inactivation of a target protein (e.g. Crh11) increased adherence (Table 2). Many of the proteins that contribute to adherence (Cht1, Cht3, Eng1, Pga4, Phr1, Phr2, Pir1, and Ssr1) also play roles in cell wall remodeling and therefore their inactivation might affect adherence indirectly possibly via cell wall defects. Nevertheless, these observations reinforce the view that carbon source affects virulence traits in part via changes in the cell wall proteome and secretome.

#### 3.2.5 Biofilm formation

Biofilm formation is dependent on adhesins that mediate the attachment of cells both to the substrate surface and cell–cell interactions [43,58]. We predicted that changes in carbon source might also affect biofilm formation and we tested the ability of cells grown on glucose or lactate to form biofilms on silicone and plastic surfaces. To parallel growth conditions used for the proteomic analysis, biofilms were incubated in minimal medium, conditions that do not promote the yeast-hyphal transition and classical biofilm formation. Instead, cells formed thin yeast-only biofilm-like structures. To test this unusual biofilm model, we compared the number of cells attached to the surface after 1 h and 24 h incubations. There was a significant increase in attachment over 24 h, suggesting that multilayered biofilm-like structures were formed over this period (Fig. 3C).

Lactate-grown cells displayed increased biofilm formation on both silicone and plastic surfaces (Fig. 3C and D). Cells grown on a mixture of glucose plus lactate formed biofilms of intermediate mass (Fig. 3C and D). Interestingly, a significant proportion (∼25%) of the cells recovered from the biofilm-like structures formed on plastic surfaces in the mixed medium were able to filament at 37°C, forming long pseudohyphae (Fig. 3E). Pseudohyphae were rare (<1%) or absent in the glucose- or lactate-grown biofilms (Fig. 3E). These observations correlate with the regulation of biofilm-associated proteins in lactate-grown cells (Supporting Information Table S3) and with the differential adhesion of cells grown on the different carbon sources (Fig. 3C and Supporting Information Fig. S2).

## 4 Concluding remarks

This study represents the first systematic comparison of the *C. albicans* cell wall proteome and secretome during growth on alternative carbon sources. Many secreted and cell wall protein levels were affected by growth on lactate versus glucose, a significant proportion being associated with functional categories of relevance to virulence (Supporting Information Tables S3). We observed a dramatic increase in the diversity and levels of cell wall remodeling proteins (Fig. 2, Supporting Information Table S3), which correlated with major alterations in the architecture of the cell wall [28]. Growth on lactate led to the formation of a relatively thin cell wall (Fig. 1A), associated with the differential expression of a battery of cell wall structural enzymes (Supporting Information Table S3). These alternative cross-linking and modifying enzymes probably contribute to the different properties of the cell wall during growth on glucose or lactate thereby affecting the elasticity of the cell wall and the resistance to osmotic, cell wall stresses, and antifungal drugs [28].

Growth on lactate also led to a different profile of adhesion-mediating proteins at the cell surface (Supporting Information Table S3). These proteins might promote the adherence of *C. albicans* in poor nutrient niches. Lactate-grown cells exhibited stronger adhesion forces [28] and all of the wild-type strains tested adhered better to plastic surfaces when grown on lactate rather than glucose (Fig. 3A and B). Lactate-grown cells formed yeast-only biofilm-like structures that were more resilient to washing than those formed by glucose-grown cells (Fig. 3C and D). This was consistent with the expression of several biofilm-related proteins in their cell wall proteome and secretome (Supporting Information Table S3). Cells grown on a mixture of glucose plus lactate displayed increased adherence and biofilm formation compared to glucose-grown cells (Fig. 3C and D) and displayed trails of adhesins between cells via their mannan fibrils (Supporting Information Fig. S2). This suggests that the availability of complex mixtures of carbon sources in certain host niches might enhance adherence and biofilm formation in vivo.

Growth on the nonfermentative carbon source led to the up-regulation of several mating and opaque-specific proteins (Supporting Information Table S3). However, this characteristic was lost in cells grown on the mixed medium (Supporting Information Tables S1 and S2). This is consistent with published reports indicating that metabolic adaptation is closely associated with mating and white-opaque switching [76,77]. Similarly, a set of metabolic and niche-specific proteins (Supporting Information Table S3) were induced in the lactate condition but not in the mixed medium cultures (Supporting Information Tables S1 and S2). Our phenotypic analyses confirmed that the induction of some cell wall integrity proteins contributes to the increased resistance displayed by lactate-grown cells (Tables 1 and 2). For over 74% of the phenotypes observed, the enrichment of a protein in one condition (glucose or lactate) correlated with a defect in the resistance or adherence of the corresponding mutant under that condition (63/85 correlate, 22/85 do not correlate, seven were carbon source independent). This confirmed our prediction that defects in proteins highly expressed in one condition will be reflected in stronger phenotypic effects under that condition. Consequently, we identified a core set of cell wall and secreted proteins (e.g. Als1, Crh11, Op4, Pga4, Pga31, Phr1, Phr2, Sap9, Ssr1) that influence the sensitivity of *C. albicans* to antifungals, cell wall, and osmotic stressors in a carbon source- specific manner.

These findings are relevant to our understanding of *C. albicans* growth and survival in the host, and hence to *C. albicans* pathogenicity. Our data suggest that the differential nutrient availability within diverse host niches is likely to have a significant impact upon the cell surface of the *C. albicans* cells in these niches, which in turn will influence their ability to counteract local chemical insults and pharmacological interventions. This work opens an important aspect of fungal pathobiology that has been largely ignored to date. The next challenge will be to define exactly which carbon sources are assimilated by *C. albicans* cells in vivo during commensalism, mucosal infection, and systemic candidiasis, and how this affects the dynamic responses of these cells to constantly fluctuating host microenvironments during disease establishment and progression.

## Abbreviations

ALS: agglutinin-like sequence
CFU: colony-forming units
CFW: calcofluor white
CR: Congo Red
GI: gastrointestinal
GPI: glycosylphosphatidylinositol

## Notes

Disclaimer: Supplementary materials have been peer-reviewed but not copyedited.

**Figure S1** Relative Mkc1 and Cek1 activation levels in glucose- and lactate-grown RM1000 cells as revealed by western blotting with phospho-specific antibodies. (A) and (B) show two independent experiments which are representative of six biological replicates. **Figure S2**. TEM images of *C. albicans* RM1000 cells grown on glucose plus lactate showing trails of mannan fibrils between cells (scale bar, 0.2 μm). This phenotype was observed in at least half of the cells in the section, n>50. **Figure S3**. Significant phenotypes observed in response to cell wall stress. The resistance of selected mutants to Calcofluor White (A, 200 μg/ml) and Congo Red (B, 300 μg/ml) were analysed. Control plates without stress are shown on the left panels. The appropriate parental control strain is displayed above each set of mutants (blue outline). Carbon source dependent phenotypes that fit or do not fit the predictions based on differential protein expression patterns are marked with ticks or “x”, respectively. Only consistent differences between wild type and mutant strains are shown, pictures being representative of three independent experiments. **Table S1**. Relative quantification of the wall proteins of *C. albicans* cells grown in glucose, lactate or a mix of the two **Table S2**. Relative quantification of the secretome of *C. albicans* cells grown in glucose, lactate or a mix of the two **Table S3**. Main functional categories of proteins regulated by carbon source **Table S4**. List of all *C. albicans* strains used in this study. **Table S5**. Detailed information on peptide identifications of the wall proteome. **Table S6**. Detailed information on peptide identifications of the secretome.
