# Supplementary material for: Carbon source-induced reprogramming of the cell wall proteome and secretome modulates the adherence and drug resistance of the fungal pathogen Candida albicans
Source: Proteomics. 2012 Oct 29;12(21):3164–79. doi: 10.1002/pmic.201200228 (PMC3569869; doi:10.1002/pmic.201200228)
Supplement: Supplementary file 1 — Figure S1 Relative Mkc1 and Cek1 activation levels in glucose- and lactate-grown RM1000 cells as revealed by western blotting with phospho-specific antibodies. (A) and (B) show two independent experiments which are representative of six biological replicates. Figure S2. TEM images of C. albicans RM1000 cells grown on glucose plus lactate showing trails of mannan fibrils between cells (scale bar, 0.2 μm). This phenotype was observed in at least half of the cells in the section, n>50. Figure S3. Significant phenotypes observed in response to cell wall stress. The resistance of selected mutants to Calcofluor White (A, 200 μg/ml) and Congo Red (B, 300 μg/ml) were analysed. Control plates without stress are shown on the left panels. The appropriate parental control strain is displayed above each set of mutants (blue outline). Carbon source dependent phenotypes that fit or do not fit the predictions based on differential protein expression patterns are marked with ticks or “x”, respectively. Only consistent differences between wild type and mutant strains are shown, pictures being representative of three independent experiments. Table S1. Relative quantification of the wall proteins of C. albicans cells grown in glucose, lactate or a mix of the two Table S2. Relative quantification of the secretome of C. albicans cells grown in glucose, lactate or a mix of the two Table S3. Main functional categories of proteins regulated by carbon source Table S4. List of all C. albicans strains used in this study. Table S5. Detailed information on peptide identifications of the wall proteome. Table S6. Detailed information on peptide identifications of the secretome. [file pmic0012-3164-SD1.pdf]

PROTEOMICS

Supporting Information for Proteomics

DOI 10.1002/pmic.201200228

Iuliana V. Ene, Clemens J. Heilmann, Alice G. Sorgo, Louise A. Walker, Chris G. de Koster, Carol A. Munro, Frans M. Klis and Alistair J. P. Brown

Carbon source-induced reprogramming of the cell wall proteome and secretome modulates the adherence and drug resistance of the fungal pathogen *Candida albicans*

© 2012 WILEY-VCH Verlag GmbH & Co. KGaA, Weinheim

Supporting information

Figure S1. Relative Mkc1 and Cek1 activation levels in glucose- and lactate-grown RM1000 cells as revealed by western blotting with phospho-specific antibodies. (A) and (B) show two independent experiments which are representative of six biological replicates.

Figure S2. TEM images of *C. albicans* RM1000 cells grown on glucose plus lactate showing trails of mannan fibrils between cells (scale bar, 0.2 µm). This phenotype was observed in at least half of the cells in the section, n>50.

Figure S3. Significant phenotypes observed in response to cell wall stress. The resistance of selected mutants to Calcofluor White (A, 200 µg/ml) and Congo Red (B, 300 µg/ml) were analysed. Control plates without stress are shown on the left panels. The appropriate parental control strain is displayed above each set of mutants (blue outline). Carbon source dependent phenotypes that fit or do not fit the predictions based on differential protein expression patterns are marked with ticks or "x", respectively. Only consistent differences between wild type and mutant strains are shown, pictures being representative of three independent experiments.

Table S1. Relative quantification of the wall proteins of *C. albicans* cultures grown in glucose, lactate or a mix of the two. Proteins were identified using MASCOT allowing two miscleavages and a tolerance of 0.3 Da ($P < 0.05$). 3-4 independent biological samples for each condition were subjected to two MS/MS runs (6-8 runs). All identified proteins were subjected to signal peptide prediction using SignalP3.0 and prediction of a GPI-anchor sequence using the BIG-PI fungal predictor. For the semi-quantitative analysis, the number

of peptides detected for each identified protein was divided by the total number of identified peptides in the respective biological replicate and multiplied by 100 to obtain the % spectral count. Spectral counts for the three growth conditions were compared using two tailed, two sample Student's t-test with a significance cut-off of 0.05. The % spectral counts were then averaged between the different biological replicates and used to calculate fold changes between the three conditions. In order to generate fold ratios between conditions for those proteins that were exclusive to one growth condition, the minimum % spectral count (one identified peptide) was used as a substitute for the growth condition in which that protein was below detection levels. Consequently, the final fold change received the "less than" (<) or "more than" (>) symbols. Numbers in bold indicate significant changes in the lactate samples with respect to glucose-grown cultures, $P < 0.05$. Stars (*) mark proteins which were included in subsequent functional analyses.

Table S2. Relative quantification of the secretome of *C. albicans* cultures grown in glucose, lactate or a mix of the two. Proteins were identified using MASCOT allowing two miscleavages and a tolerance of 0.3 Da ($P < 0.05$). 3-4 independent biological samples for each condition were subjected to two MS/MS runs (6-8 runs). All identified proteins were subjected to signal peptide prediction using SignalP3.0 and prediction of a GPI-anchor sequence using the BIG-PI fungal predictor. For the semi-quantitative analysis, the number of peptides detected for each identified protein was divided by the total number of identified peptides in the respective biological replicate and multiplied by 100 to obtain the % spectral count. Spectral counts for the three growth conditions were compared using two tailed, two sample Student's t-test with a significance cut-off of 0.05. The % spectral counts were then averaged between the different biological replicates and used to calculate fold changes between the three conditions. In order to generate fold ratios between conditions for those

proteins that were exclusive to one growth condition, the minimum % spectral count (one identified peptide) was used as a substitute for the growth condition in which that protein was below detection levels. Consequently, the final fold change received the “less than” (<) or “more than” (>) symbols. Numbers in bold indicate significant changes in the lactate samples with respect to glucose-grown cultures, $P < 0.05$. Stars (*) mark proteins which were included in subsequent functional analyses.

Table S3. Main functional categories regulated by carbon source. Proteins marked in the table are those involved in cell wall structure and remodelling, adherence, regulated during biofilm formation, mating and phenotypic switching, metabolism and niche specific proteins. Fold changes were calculated as the fraction between the relative % spectral counts identified in the wall proteome or secretome of lactate- relative to glucose-grown cultures (L/G). Numbers in bold indicate significant changes in the lactate samples with respect to glucose-grown cultures, $P < 0.05$. Stars (*) mark proteins which were included in subsequent functional analyses.

Table S4. List of all *C. albicans* strains used in this study.

Table S5. Detailed information on peptide identifications of the wall proteome.

Table S6. Detailed information on peptide identifications of the secretome.

Figure S1.

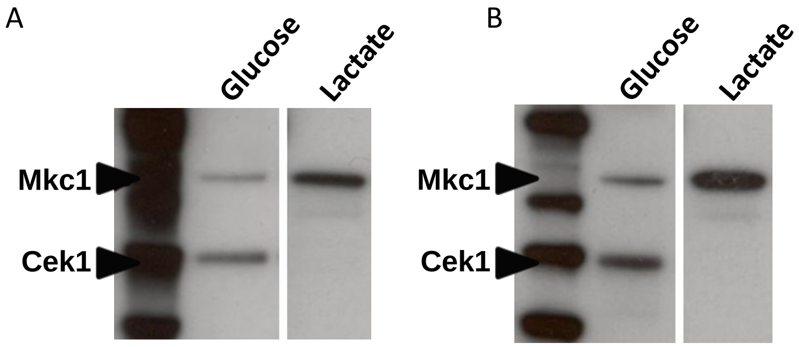

Figure S2.

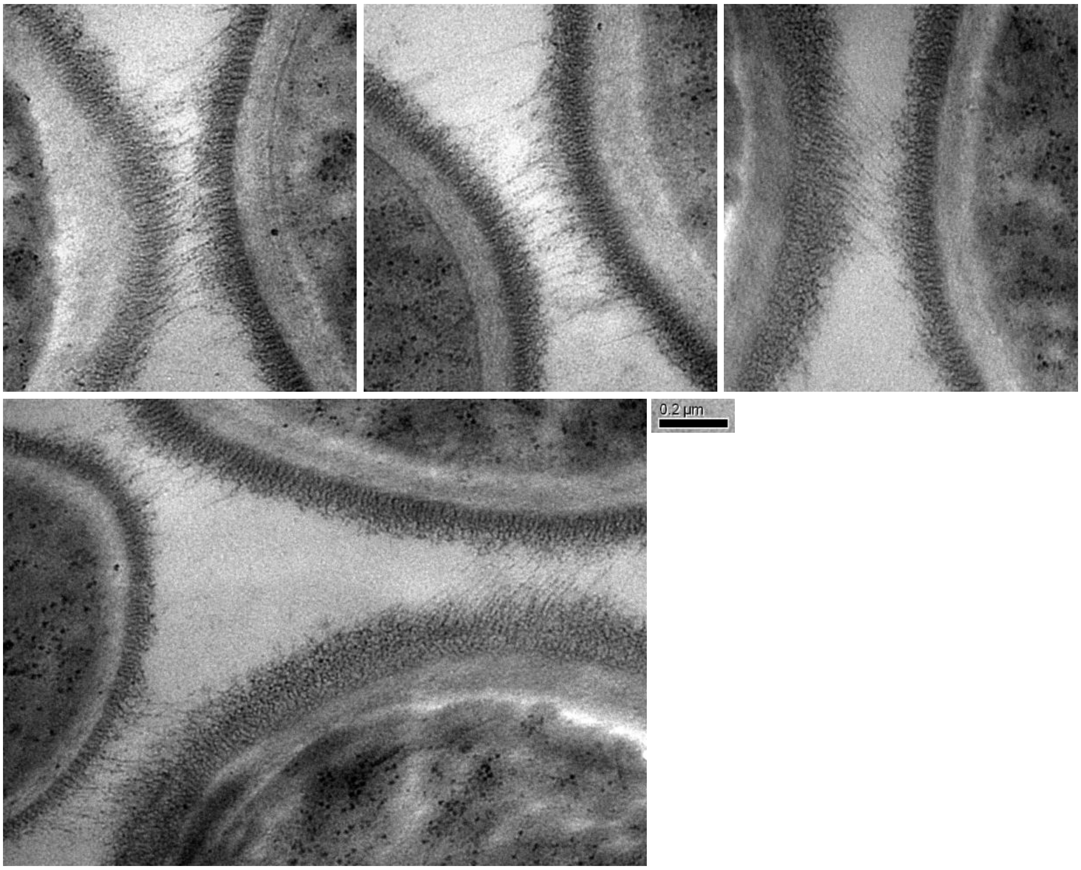

Figure S3.

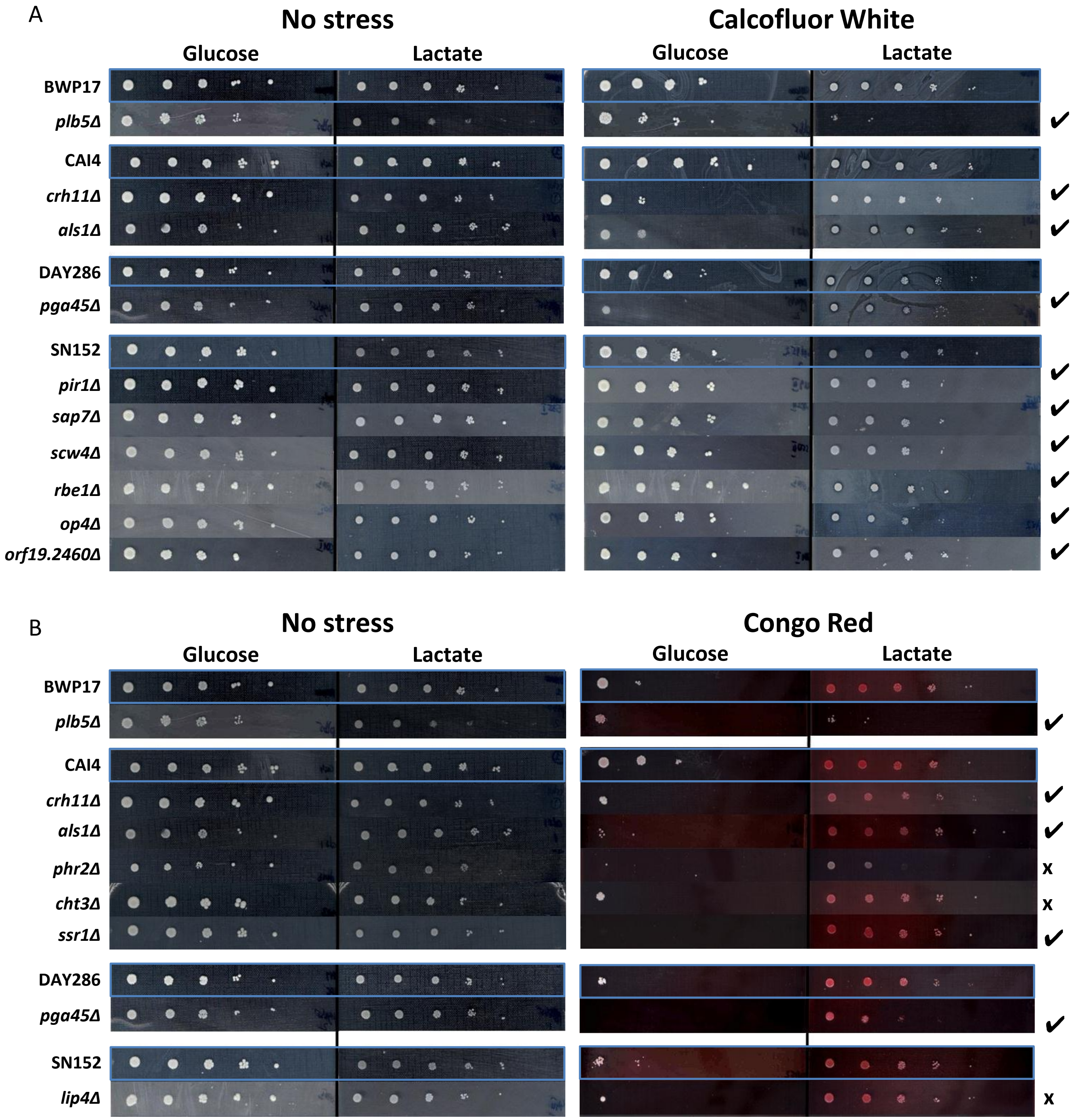
A
No stress
Calcofluor White
Glucose
Lactate
Glucose
Lactate
BWP17
plb5Δ
CAI4
crh11Δ
als1Δ
DAY286
pga45Δ
SN152
pir1Δ
sap7Δ
scw4Δ
rbe1Δ
op4Δ
orf19.2460Δ
B
No stress
Congo Red
Glucose
Lactate
Glucose
Lactate
BWP17
plb5Δ
CAI4
crh11Δ
als1Δ
phr2Δ
cht3Δ
ssr1Δ
DAY286
pga45Δ
SN152
lip4Δ
x
x
x

Table S1. Relative quantification of the wall proteins of *C. albicans* cells grown in glucose, lactate or a mix of the two

**PROTEOME**<br>**Protein**	**Glucose**<br>**(n=3)**	**Glucose + Lactate**<br>**(n=4)**	**Lactate**<br>**(n=4)**	**Fold change (G + L)/G**	**Fold change L/G**	**Tested**
Lactate exclusive						
**Als2**	n.d.	2.20	1.35	>8.28	**>5.07**	
**Pga31**	n.d.	n.d.	5.01		**>18.83**	*
**Phr1**	n.d.	n.d.	1.87		**>7.03**	*
**Plb5**	n.d.	n.d.	0.51		>1.92	*
**Sap9**	n.d.	n.d.	3.67		**>13.78**	*
**Sun41**	n.d.	n.d.	0.27		>1	
**Tos1**	n.d.	n.d.	0.53		>2	
**Yps7**	n.d.	0.27	1.30	>1	**>4.89**	*
Increased with lactate (>1.5)						
**Pga4**	1.65	6.24	6.06	**3.78**	**3.67**	*
**Phr2**	3.69	4.35	6.60	1.18	**1.79**	*
No significant change						
**Asl4**	6.11	3.77	5.59	0.62	0.91	
**Cht2**	10.53	7.87	8.16	0.75	**0.78**	
**Coi1**	n.d.	0.52	n.d.	>1.94		
**Ecm33**	12.60	8.41	9.51	**0.67**	**0.76**	
**Mp65**	4.86	9.66	6.35	**1.99**	1.31	
**Pir1**	4.07	4.58	5.00	1.13	1.23	*
**Rbt1**	n.d.	0.27	n.d.	>1		
**Rbt5**	2.44	2.16	2.11	**0.89**	**0.86**	*
**Rhd3**	10.59	14.36	11.06	**1.36**	1.04	
**Sod4**	1.22	1.37	1.02	1.13	0.84	
**Utr2**	4.86	5.37	3.93	1.11	0.81	
**Ywp1**	2.86	2.70	3.45	0.95	1.21	
Decreased with lactate (<0.66)						
**Als1**	9.33	6.22	0.27	**0.67**	**0.03**	*
**Crh11**	15.47	12.41	8.74	**0.80**	**0.56**	*
**Ssr1**	3.66	2.96	1.30	0.81	**0.35**	*
Glucose exclusive						
**Pga45**	0.41	n.d.	n.d.	<0.65	<0.65	*

n = number of biological replicates   bold = significant changes   n.d. = not detected

Table S2. Relative quantification of the secretome of *C. albicans* cells grown in glucose, lactate or a mix of the two

SECRETOME<br>Protein	Glucose<br>(n=3)	Glucose + Lactate<br>(n=4)	Lactate<br>(n=4)	Fold change (G + L)/G	Fold change L/G	Tested
Lactate exclusive						
**Asl4**	n.d.	n.d.	0.48		>2.26	
**Cht1**	n.d.	n.d.	0.67		>3.18	*
**Gca1**	n.d.	0.22	4.30	>1.05	**>20.32**	
**Ilv5**	n.d.	n.d.	0.21		>1	
**Lip4**	n.d.	n.d.	0.21		>1	*
**Op4**	n.d.	n.d.	0.67		>3.18	*
**Orf19.1239**	n.d.	n.d.	0.92		>4.36	*
**Orf19.2460**	n.d.	n.d.	0.25		>1.18	*
**Orf19.3499**	n.d.	n.d.	0.27		>1.26	
**Pry1**	n.d.	n.d.	0.46		>2.18	*
**Sap7**	n.d.	n.d.	2.58		**>12.20**	*
**Scw4**	n.d.	n.d.	0.64		>3	*
Increased with lactate (>1.5)						
**Cht3**	5.07	5.71	7.62	1.13	**1.50**	*
**Pir1**	1.75	1.94	2.83	1.11	1.62	*
**Rbe1**	0.84	0.27	1.61	0.32	1.92	*
**Xog1**	0.84	5.81	4.25	**6.91**	**5.06**	
No significant change						
**Als1**	n.d.	0.49	n.d.	>2.34		*
**Dag7**	5.44	4.34	4.55	0.80	0.84	
**Ecm33**	2.71	0.44	1.91	**0.16**	0.71	
**Eng1**	3.34	4.18	2.83	1.25	0.85	*
**Mp65**	10.43	12.49	12.27	**1.20**	1.18	
**Pga45**	n.d.	2.88	n.d.	**>13.59**		*
**Plb4.5**	0.28	n.d.	0.21	<0.76	0.76	
**Rhd3**	3.84	1.67	3.51	**0.43**	0.91	
**Scw11**	5.59	8.28	4.98	**1.48**	0.89	
**Ssr1**	3.27	2.62	3.39	0.80	1.04	*
**Sun41**	8.06	10.06	6.22	**1.25**	**0.77**	
**Tos1**	10.87	10.97	13.77	1.01	1.27	
**Utr2**	3.27	3.07	3.83	0.94	**1.17**	
Decreased with lactate (<0.66)						
**Bgl2**	5.82	3.04	1.70	0.52	**0.29**	
**Cht2**	4.20	5.89	1.42	1.40	**0.34**	
**Msb2**	2.63	0.27	0.23	**0.10**	**0.09**	*
**Rbt4**	2.03	0.69	0.23	0.34	**0.11**	*
**Sim1**	10.15	10.65	6.65	1.05	**0.66**	
**Ywp1**	1.47	2.63	0.21	**1.79**	**0.14**	
Glucose exclusive						
**Fet3**	0.33	n.d.	n.d.	<0.65	<0.65	*
**Nup**	2.37	0.93	n.d.	**0.39**	**<0.09**	
**Orf19.4886**	0.29	n.d.	n.d.	<0.72	<0.72	
**Pga4**	0.28	n.d.	n.d.	<0.76	<0.76	*
**Phr2**	0.28	n.d.	n.d.	<0.76	<0.76	*
**Rbt5**	0.33	n.d.	n.d.	<0.65	<0.65	*

n = number of biological replicates   bold = significant changes   n.d. = not detected

Table S3. Main functional categories of proteins regulated by carbon source

Protein	Tested	Fold change L/G		Function	Cell wall integrity	Adherence	Biofilm formation	Mating regulated	Metabolism/ niche specific
		Proteome	Secretome						
Als1	*	**0.03**	-	adhesin, biofilm-induced		x	x		
Als2		**>5.07**	-	adhesin, biofilm-induced		x	x		
Als4		0.91	>2.26	adhesin		x			
Bgl2		-	0.29	glucosyltransferase	x				
Cht1	*	-	>3.18	chitinase	x				
Cht2		**0.78**	**0.34**	chitinase	x				
Cht3	*	-	**1.5**	chitinase	x				
Crh11	*	**0.56**	-	glucan-chitin crosslinker, upregulated in low oxygen conditions	x				
Dag7		-	0.84	secretory protein				x	
Ecm33		**0.76**	0.71	adhesion, cell wall architecture	x	x			
Eng1	*	-	0.85	endo-1,3-β-glucanase, biofilm induced, pheromone regulated	x		x	x	
Gca1		-	**>20.3**	glucoamylase, biofilm induced, possible adhesin, oral infection, nutrient regulation		x	x		x
Ilv5		-	>1	amino acid starvation, Gcn4 regulated, biofilm induced			x		x
Lip4	*	-	>1	secreted lipase					x
Mp65		1.31	1.18	endo-1,3-β-glucanase, biofilm, adhesion	x	x	x		
Msb2	*	-	**0.09**	adhesin, cell wall damage sensor	x	x			
Op4	*	-	>3.18	opaque specific, unknown function				x	
Orf19.2460	*	-	>1.18	pheromone regulated				x	
Orf19.4886		-	<0.72	adhesin-like		x			
Pga4	*	**3.67**	<0.76	glucanosyltransferase, upregulated in oral infection	x				x
Pga31	*	**>18.8**	-	regulated upon white-opaque switching and cell wall regeneration	x			x	
Pga45	*	<0.65	-	GPI-CWP, Hog1 induced, biofilm induced			x		
Phr1	*	**>7.03**	-	glucanosyltransferase, high pH induced, adhesion to epithelial cells	x	x			
Phr2	*	**1.79**	<0.76	glucanosyltransferase, low pH induced	x				
Pir1	*	1.23	1.62	crosslinking β-1,3-glucans	x				

Plb4.5		-	0.76	phospholipase B, Hog1-induced, repressed during cell wall regeneration	**x**				
Plb5	*	>1.92	-	phospholipase B					**x**
Pry1	*	-	>2.18	opaque specific				**x**	
Rbe1	*	-	1.92	Rim101, Efg1 regulated, biofilm induced			**x**		
Rbt1		only in G+L		filamentous growth, mating, adhesion, biofilm formation		**x**	**x**	**x**	
Rbt4	*	-	**0.11**	Efg1 regulated, pheromone regulated				**x**	
Rbt5	*	**0.86**	<0.65	hemoglobin utilisation, induced by iron starvation, Bcr1 regulated			**x**		
Sap7	*	-	**>12.2**	aspartyl protease, oral and vaginal infection, pheromone regulated				**x**	**x**
Sap9	*	**>13.8**	-	aspartyl protease, yapsin, adhesion to epithelial cells, biofilm formation		**x**	**x**		
Scw4	*	-	>3	white-opaque switching regulated, biofilm induced			**x**	**x**	
Scw11		-	0.89	1,3-β-glucanase, expression greater in high iron	**x**				
Sim1		-	**0.66**	adhesin-like, cell wall maintenance, redundant with Sun41	**x**	**x**			
Sod4		0.84	-	super oxide dismutase, white-opaque switching regulated				**x**	
Ssr1	*	**0.35**	1.04	β-glucan associated, cell wall structure	**x**				
Sun41		>1	**0.77**	glycosidase, cell wall architecture, adhesion and biofilm formation	**x**	**x**	**x**		
Tos1		>2	1.27	pheromone regulated, described as similar to alpha agglutinin anchor subunit				**x**	
Utr2		0.81	**1.17**	glucan-chitin crosslinker, adhesion to epithelial cells	**x**	**x**			
Xog1		-	**5.06**	exo-1,3-β-glucanase, induced under low iron conditions	**x**				
Yps7	*	**>4.89**	-	endopeptidase, mutants show increased sensitivity to alpha pheromone				**x**	
Ywp1		1.21	**0.14**	mutation causes increased adhesion and biofilm formation		**x**	**x**		

Table S4. List of *C. albicans* strains used in this study

Strain	Name	Genotype	Source
CAI4		*ura3Δ::λimm434/ura3Δ::λimm434*	(Fonzi, Irwin 1993)
CAI4 + CIp10		*ura3Δ::λimm434/ura3Δ::λimm434 RPS1: Clp10 (URA3)*	(Murad et al. 2000)
RM1000		*ura3Δ::λimm434/ura3Δ::λimm434, his1::hisG/his1::hisG*	(Negredo et al. 1997)
RM1000 + CIp20		*ura3Δ::λimm434/ura3Δ:: λimm434, his1::hisG/his1::hisG, RSP1::Clp20 (URA3, HIS1)*	(Smith et al. 2004)
BWP17		*ura3Δ::λimm434/ura3Δ::λimm434, his1::hisG/his1::hisG, arg4::hisG/arg4::hisG*	(Wilson, Davis & Mitchell 1999)
BWP17 + CIp30		*ura3Δ::λimm434/ura3Δ::λimm434, his1::hisG/his1::hisG, arg4::hisG/arg4::hisG, RPS1::CIp30 (URA3, HIS1, ARG4)*	(Davis et al. 2000)
DAY286		*ura3Δ::λimm434/ura3Δ::λimm434, his1::hisG/his1::hisG, pARG4::URA3::arg4::hisG/arg4::hisG*	(Davis et al. 2000)
SN152		*arg4Δ /arg4Δ , leu2Δ/leu2Δ, his1Δ /his1Δ, URA3/ura3Δ::λimm434, IRO1/iro1Δ::λimm434*	(Noble, Johnson 2005)
als1Δ	CAYC2	*als1Δ::hisG-URA3-hisG/als1Δ::hisG, ura3Δ::λimm434/ura3Δ::λimm434*	(Fu et al. 2002)
cht1Δ		*ura3Δ::λimm434/ura3Δ::λimm434, cht1::hisG/cht1::hisG, pARG4::URA3::arg4::hisG/arg4::hisG*	(Plaine et al. 2008)
cht3Δ	SPY24	*cht3Δ::hisG-URA3-hisG/cht3Δ::hisG*	(Selvaggini et al. 2004)
crh11Δ	GPY80	*crh11Δ::hisG-URA3-hisG/crh11Δ::hisG*	(Pardini et al. 2006)
eng1Δ	#564	*eng1Δ::LEU2/eng1Δ::HIS1, arg4Δ/arg4Δ, leu2Δ/leu2Δ, his1Δ/his1Δ, URA3/ura3Δ::λimm434, IRO1/iro1Δ::λimm443*	(Noble et al. 2010)
fet3Δ	#712	*fet3Δ::LEU2/fet3Δ::HIS1, arg4Δ/arg4Δ, leu2Δ/leu2Δ, his1Δ/his1Δ, URA3/ura3Δ::λimm434, IRO1/iro1Δ::λimm444*	(Noble et al. 2010)
lip4Δ	#44	*lip4Δ::LEU2/lip4Δ::HIS1, arg4Δ/arg4Δ, leu2Δ/leu2Δ, his1Δ/his1Δ, URA3/ura3Δ::λimm434, IRO1/iro1Δ::λimm435*	(Noble et al. 2010)
msb2Δ	#372	*msb2Δ::LEU2/msb2Δ::HIS1, arg4Δ/arg4Δ, leu2Δ/leu2Δ, his1Δ/his1Δ, URA3/ura3Δ::λimm434, IRO1/iro1Δ::λimm439*	(Noble et al. 2010)
op4Δ	#104	*op4Δ::LEU2/op4Δ::HIS1, arg4Δ/arg4Δ, leu2Δ/leu2Δ, his1Δ/his1Δ, URA3/ura3Δ::λimm434, IRO1/iro1Δ::λimm436*	(Noble et al. 2010)
orf19.1239Δ	#24	*orf19.1239Δ::LEU2/orf19.1239Δ::HIS1, arg4Δ/arg4Δ, leu2Δ/leu2Δ, his1Δ/his1Δ, URA3/ura3Δ::λimm434, IRO1/iro1Δ::λimm434*	(Noble et al. 2010)
orf19.2460Δ	#501	*orf19.2460Δ::LEU2/orf19.2460Δ::HIS1, arg4Δ/arg4Δ, leu2Δ/leu2Δ, his1Δ/his1Δ, URA3/ura3Δ::λimm434, IRO1/iro1Δ::λimm440*	(Noble et al. 2010)
pga4Δ		*ura3Δ::λimm434/ura3Δ::λimm434, pga4::hisG/pga4::hisG, pARG4::URA3::arg4::hisG/arg4::hisG*	(Plaine et al. 2008)
pga31Δ		*ura3Δ::λimm434/ura3Δ::λimm434, pga31::hisG/pga31::hisG,*	(Plaine et al. 2008)

		pARG4::URA3::arg4::hisG/arg4::hisG	
pga45Δ		*ura3Δ::λimm434/ura3Δ::λimm434, pga45::hisG/pga45::hisG, pARG4::URA3::arg4::hisG/arg4::hisG*	(Plaine et al. 2008)
phr1Δ	CAS7	*phr1Δ::hisG/phr1Δ, ura3Δ::λimm434/ura3Δ::λimm434*	(Saporito-Irwin et al. 1995)
phr2Δ	CFM-2	*phr2Δ::hisG-URA3-hisG/phr2Δ::hisG, ura3Δ::λimm434/ura3Δ::λimm434*	(Muhlschlegel, Fonzi 1997)
pir1Δ	#219	*pir1Δ::LEU2/pir1Δ::HIS1, arg4Δ/arg4Δ, leu2Δ/leu2Δ, his1Δ/his1Δ, URA3/ura3Δ::λimm434, IRO1/iro1Δ::λimm437*	(Noble et al. 2010)
plb5Δ		*plb5Δ::hisG-URA3-hisG/plb5Δ::hisG*	M. Richard, INRA
pry1Δ	#535	*pry1Δ::LEU2/pry1Δ::HIS1, arg4Δ/arg4Δ, leu2Δ/leu2Δ, his1Δ/his1Δ, URA3/ura3Δ::λimm434, IRO1/iro1Δ::λimm441*	(Noble et al. 2010)
rbe1Δ	#1070	*rbe1Δ::LEU2/rbe1Δ::HIS1, arg4Δ/arg4Δ, leu2Δ/leu2Δ, his1Δ/his1Δ, URA3/ura3Δ::λimm434, IRO1/iro1Δ::λimm447*	(Noble et al. 2010)
rbt4Δ	#960	*rbt4Δ::LEU2/rbt4Δ::HIS1, arg4Δ/arg4Δ, leu2Δ/leu2Δ, his1Δ/his1Δ, URA3/ura3Δ::λimm434, IRO1/iro1Δ::λimm445*	(Noble et al. 2010)
rbt5Δ	BCa18-2	*rbt5Δ::hisG-URA3-hisG/rbt5Δ::hisG ura3Δ::λimm434/ura3Δ::λimm434*	(Braun et al. 2000)
sap7Δ	#282	*sap7Δ::LEU2/sap7Δ::HIS1, arg4Δ/arg4Δ, leu2Δ/leu2Δ, his1Δ/his1Δ, URA3/ura3Δ::λimm434, IRO1/iro1Δ::λimm438*	(Noble et al. 2010)
sap9Δ		*sap9Δ::hisG-URA3-hisG/sap9Δ::hisG, ura3Δ::λimm434/ura3Δ::λimm434*	(Albrecht et al. 2006)
scw4Δ	#550	*scw4Δ::LEU2/scw4Δ::HIS1, arg4Δ/arg4Δ, leu2Δ/leu2Δ, his1Δ/his1Δ, URA3/ura3Δ::λimm434, IRO1/iro1Δ::λimm442*	(Noble et al. 2010)
ssr1Δ	30542-2	*ssr1Δ::hisG-URA3-hisG/ssr1Δ::hisG ura3::λimm434/ura3::λimm434*	(Garcera et al. 2005)
yps7Δ	#988	*yps7Δ::LEU2/yps7Δ::HIS1, arg4Δ/arg4Δ, leu2Δ/leu2Δ, his1Δ/his1Δ, URA3/ura3Δ::λimm434, IRO1/iro1Δ::λimm446*	(Noble et al. 2010)

Table S5. Detailed information on peptide identifications of the wall proteome.

			Glucose			Glucose + Lactate				Lactate			
**Protein**	**Residues**	**Peptides identified**	**R1**	**R2**	**R3**	**R1**	**R2**	**R3**	**R4**	**R1**	**R2**	**R3**	**R4**
Als fam	237-254	GLNDWNYPVSSESFSYTK	1	1	2	2	1						
Als1	19-41	TITGVFDSFNSLTWSNAANYAFK		1	1		1	1					
	77-92	YTTSQTSVDLTADGVK	1	1	1								
	124-149	AFGTVTLPIAFNVGGTGSSTDLEDSK	1	1	2								
	150-175	[CFTAGTNTVTFNDGDKDISIDVEFEK]	1										
	176-188	STVDPSGYLYASR	1	1	1	2	2	1	2				1
	189-195	VMPSLNK	1	1	2	1							
	255-264	TCTSNGIQIK	2	2	2	1	1	2	2				
	303-311	SQSKPFTLR				2	1	2	2				
Als2	19-34	VITGVFNSFDSLTWTR								1		1	1
	35-41	AGNYAYK						1					
	93-111	YATCQFYSGEEFTTFSSLK						1					
	112-123	CTVSNTLTSSIK										1	
	153-166	AGTNTVTFNDGDKK						1	2				
	291-310	YTNDYACVGSSLQSKPFNLR						2	1				1
Asl4	19-41	VITGIFDSFNSLTWTNAASYSYR											2
	77-91	FITDQTSIDLVADGR	2	1	1	2	2	1	2	2	1	1	2
	92-123	TYATCNLNSAEEFTTFSSVSCTVTTTMTADTK								1	2	2	
	255-284	TCTSTSVLVTYQNVPAGYRPFVDAYVSATR	2	1	2	1	2					1	1
	285-291	VSSYAMR	2	2	2	2	2			2		2	2
Cht2	92-98	TCQSLGK	2	2	2					2	2	2	2
	99-121	TVLLSLGGGVGDYGFSDVASATK	1	1	2								
	122-129	FADTLWNK						1					
	130-168	FGAGEDPERPFDDAVVDGFDFDIEHGGATGYPELATALR			1	1				2	1		1
	178-200	NYFLSAAPQCPYPDASLGDLLSK	1			2		1	1		1	1	
	201-228	VPLDFAFIQFYNNYCSINGQFNYDTWSK						1				1	1
	229-236	FADSAPNK	2	2	2	2	2	2	2	2	2	2	2
	240-258	LFVGVPATSNIAGYVDTSK				1	2	1	1	1	2	1	2
	259-267	LSSAIEEIK	2	2	2		1	2	2				
	291-299	GENFVVQVK			2		1	1	2	1			
Coi1	144-152	YSCLLDVMK				2							
Crh11	29-45	SSDCSPVPALGSSFLEK	1			1	2	1	1				
	46-59	FDNGLGPHFESLKK	1	1	1			2	1	2	2	2	1
	60-75	QGTIDSGSNGLSLTMK		1	1					1	1	1	
	77-84 [78-84]	FDNPSFK [RFDNPSFK]	2	2	2	2	2	2	2				
	85-93	SNFYIMFGR	2	2	2	2	2	1	2	1			1
	148-158	GGYHDIANPLK	2	2	2	2	1	1		2		2	1
	159-169	DYHTYVIDWTK	2	2	2	2	1			2		1	
	170-182	DAVTWSVDGSVIR	1	1	1								
	236-246	SVLVADYSSGK				1	1	2	2		2		1
	247-261	QYSYSDQSGSWESIK	2	2	1	2	2	2	2	2	2	2	2
	271-279	YDQAQDDIK				2				1			1

Ecm33	139-168	TGLTAGITSAESVVISDTGLSSLTGINVFK	1	1	2	1	2	2	1	2	1	2	1
	171-202	VFNVNNNDNIDTIDSGLQEVTDTIDISYNAEK											1
	246-262	VELAELTSIGNSLTINK	2	2	1	2	2			2	2	2	2
	263-273	NDDLTELDFPK									1	2	2
	276-290	TIGGALQISDNSELR	2	2	2	2	2	1	2	2	2	2	2
	291-297	SFSGFPK	1	2	2		1						
	322-329	VSGGFILK	2	1	2		1	2	2				
	334-341	LSCSAFNK	2	2	2	2	2	2	2	2	2	2	1
	342-349	LNTNGDIK											1
Mp65	128-141	GITYSPYSDNGGCK	1			2	1		2	2		1	1
	142-160	SESQIASEIAQLSGFDVIR				1	1			1			
	161-174	LYGVDCDQVSAVLK		2	2		2				1	1	
	234-240 [234-241]	AYVDEGR [AYVDEGRK]				2		1	2				1
	302-309 [302-310]	VWTACSGK			1	2	2	2	2	2	2	2	2
	323-333	GDSNGLAVPSK				1							
	334-344	SNQQAAISSIK	2	2	2	2	2	2	2	2	2	2	2
	363-371 [363-378]	ADGPYNAEK [ADGPYNAEKYGIYSN] (C-term)				2		1	2				
Pga4	69-79	YFQELGINTIR		2		1	1	2	2	2	2	1	2
	99-116	AGIYVILDVNTPHSSITR	1	1		2	2	2	2				
	184-198	TIPVGYSAASVDEYR						1					
	199-214	LPSGLYFNCGDDDMAR								2	2	2	1
	215-241	IDMYGINDYSWCGDASMTTSQYSQQMK											1
	323-332 [321-332]	NPSGDGGYLK [TKNPSGDGGYLK]				2	2	2	2	2	2	2	2
Pga31	102-122	HEGAALNYLFLAAPGVAENLK								1	1	1	1
	143-162	QPLNVGNTVLQLGGSGDGTK								2	2	2	2
	184-193	NINDPYNYSK								1	2	2	1
	201-209	GGDGAIPIK								1			
Phr fam		SNTDASAFVK	2	1	2	2	2	2	2	2	2	2	2
Phr1	216-230	VAIADYFSCGSLDDR								2	1	1	
	231-244	ADFFGINMYEWCGK										2	
	438-452	LSYVLNQYYLDQDKK											1
Phr2	89-125	VYALDTSQDHTECMQMLQDAGIYVIADLSQPDESINR								2	1		1
	126-137	DDPSWDLDLFER								1	2	2	1
	191-205	SIPVGYSANDDSAIR	2			2	1	2	2	1	1	1	2
	239-250	ASGYESATNDYK								1			
	251-269	NLGIPIFFSEYGCNEVRPR	2	2	2		2	1	2	2	2	2	2
	424-441	DKLSFVMNLYYEQNKESK	1					2	2			1	
Pir1	249-266	ACSSANNLEMTLHDSVLK	1	2	2	1	1	2	2	2	2	2	2
	272-301	WGAIVANHQFQFDGPIPQAGTIYSAGWSIK	2	2	1	2	2	1	2	2	2	2	2
	302-331	DGYLYLGDSNIFYQCLSGDFYNLYDENVAK				2	2				1	1	1

Plb5	250-266	STIWQLQDSILNPSGMR									1		
	270-284	TIAYYYGLAQAVQAK											1
Rbt1	245-254	TLGLLLGLLK							1				
CFEM fam	47-56	IYDQLPECAK	1	2	2			2	2				
Rbt5	61-92	QSTSSTPCPYWDTGCLCVMPQFAGAVGNCVAK	2	2	2	2	2	2	2	2	2	2	2
Rhd3	31-39 [26-39]	VDGLGLYSK [SDDSKVDGLGLYSK]	1	2		2	2	1					
	40-52	HEGAAIDYLFLGK						2	2				
	64-71 [59-71]	QIFQELK [YDDEKKQIFQELK]	1	1	2		1			2	2	2	2
	73-78	TSSITVR	2	2	2	2	2	2	2	2	2	2	2
	79-103	QSFTLGGDVYELGATDNFIPVTINK						2	2	1	1	1	
	104-114	DGTLSFTGDDK				2	2	2	2				1
	120-126	NVNDPYR		1		2	2	1	2	2	2	2	2
	127-137	YSESEYAVSNK	2	2	2	2	2	2	2	2	2	2	1
	140-151 [139-151]	TDDSAPITIVAK [KTDDSAPITIVAK]	2	2	2	2	2	2	2	2	2	1	2
Sap9	294-306	SLYSLYLNTADAK								1	2	2	
	320-328	YQGDLVTVK								1	1	1	
	332-341	TYSQISYPVR								1	1		
	427-438	VPISDLVLQASK											1
	439-463	STCILGVMQQSSSSSYMLFGDNILR									1	2	
Sod4	28-33 [26-33]	APLVAR [GKAPLVAR]						2	1		2		2
	105-117	TPAALELGDLSGR	1	1	1			1	1				
Ssr1	41-54	CSGLNDLSCICTTK	1	1	1	2	1						
	64-79	EICPNGDADTAISAFK	2	2	2	2	2	2	2	1	2	1	1
Sun41	297-331	TDYPGTENMVIPTVVGGGSTSVITVVDQSTYYTWR											1
Tos1	231-259	DSYYTPGSTDNCVFLNYHGGSGSGVWSAK											2
Utr2	75-85	MSTFQESFDSK							1				
	137-142	YLWYGK									1		
	245-253	YDYPQTPSR	2	1	2	1	1			2	2	2	1
	254-268	IQFSLWPGGDSSNAK		2	2	2	2			1	2	1	1
	269-286	GTIEWAGGLINWDSEDIK	1		1	2	2	1	2			2	
	288-296	YGYYYAHIK		1		2	2	1	1				
Yps7	494-511	VVPSSVTGYNMLGLPFLK							1	2	2		1
Ywp1	87-93	VINVPAR	1			1	1			1			1
	94-110	NLYGAGAVPFFQVHLEK								1			2
	329-339	VITVVACDEHK	2	2	2	2	2	2	2	2	2	2	2
**Total peptide count**			**80**	**80**	**85**	**97**	**90**	**89**	**94**	**92**	**102**	**92**	**94**
contaminant peptides						1					14	2	1

Glucose

name	R1	R2	R3	average	SD	SE	CV%
Als1	0.09	0.09	0.11	0.09	0.01	0.01	6.77
Als2	n.d.	n.d.	n.d.				
Asl4	0.08	0.05	0.06	0.06	0.01	0.01	12.25
Cht2	0.10	0.09	0.13	0.11	0.02	0.01	12.05
Crh11	0.16	0.16	0.14	0.15	0.01	0.01	4.39
Ecm33	0.13	0.12	0.13	0.13	0.00	0.00	1.42
Mp65	0.04	0.05	0.06	0.05	0.01	0.01	12.70
Pga4	0.01	0.04	n.d.	0.02	0.02	0.01	40.44
Pga31	n.d.	n.d.	n.d.				
Phr1	n.d.	n.d.	n.d.				
Phr2	0.06	0.02	0.02	0.04	0.02	0.01	34.68
Pir1	0.04	0.05	0.04	0.04	0.01	0.00	10.74
Plb5	n.d.	n.d.	n.d.				
Rbt5	0.03	0.02	0.02	0.02	0.00	0.00	1.83
Rhd3	0.10	0.12	0.09	0.11	0.02	0.01	8.47
Sap9	n.d.	n.d.	n.d.				
Sod4	0.01	0.01	0.01	0.01	0.00	0.00	1.83
Ssr1	0.04	0.04	0.04	0.04	0.00	0.00	1.83
Utr2	0.04	0.05	0.06	0.05	0.01	0.01	12.70
Yps7	n.d.	n.d.	n.d.				
Ywp1	0.04	0.02	0.02	0.03	0.01	0.00	15.66
					**median CV%**		**10.74**

Glucose + Lactate

name	R1	R2	R3	R4	average	SD	SE	CV%
Als1	0.06	0.06	0.07	0.06	0.06	0.00	0.00	4.00
Als2	n.d.	n.d.	0.06	0.03	0.04	0.02	0.01	19.48
Asl4	0.05	0.07	0.01	0.02	0.04	0.03	0.01	34.27
Cht2	0.06	0.07	0.10	0.09	0.08	0.02	0.01	11.44
Crh11	0.14	0.12	0.12	0.11	0.12	0.02	0.01	6.27
Ecm33	0.07	0.11	0.08	0.07	0.08	0.02	0.01	10.82
Mp65	0.12	0.09	0.07	0.11	0.10	0.02	0.01	12.47
Pga4	0.05	0.06	0.08	0.06	0.06	0.01	0.01	9.60
Pga31	n.d.	n.d.	n.d.	n.d.				
Phr1	n.d.	n.d.	n.d.	n.d.				
Phr2	0.02	0.03	0.06	0.06	0.04	0.02	0.01	23.00
Pir1	0.05	0.06	0.03	0.04	0.05	0.01	0.00	10.63
Plb5	n.d.	n.d.	n.d.	n.d.				
Rbt5	0.02	0.02	0.02	0.02	0.02	0.00	0.00	1.98
Rhd3	0.12	0.14	0.16	0.15	0.14	0.01	0.01	4.98
Sap9	n.d.	n.d.	n.d.	n.d.				
Sod4	n.d.	n.d.	0.03	0.02	0.03	0.01	0.00	15.99
Ssr1	0.04	0.03	0.02	0.02	0.03	0.01	0.00	16.02
Utr2	0.07	0.08	0.02	0.04	0.05	0.03	0.01	24.14
Yps7	n.d.	n.d.	n.d.	0.01	0.01			
Ywp1	0.03	0.03	0.02	0.02	0.03	0.01	0.00	11.15
						**median CV%**		**11.30**

Lactate

name	R1	R2	R3	R4	average	SD	SE	CV%
Als1	n.d.	n.d.	n.d.	0.01	0.01			
Als2	0.01	n.d.	0.02	0.02	0.02	0.01	0.00	17.11
Asl4	0.05	0.03	0.07	0.07	0.06	0.02	0.01	17.41
Cht2	0.09	0.08	0.08	0.09	0.08	0.01	0.00	3.19
Crh11	0.12	0.07	0.09	0.07	0.09	0.02	0.01	13.02
Ecm33	0.09	0.08	0.11	0.11	0.10	0.01	0.01	7.78
Mp65	0.08	0.05	0.07	0.06	0.06	0.01	0.01	8.75
Pga4	0.07	0.06	0.05	0.06	0.06	0.00	0.00	4.10
Pga31	0.05	0.05	0.05	0.04	0.05	0.01	0.00	5.60
Phr1	0.02	0.01	0.03	0.01	0.02	0.01	0.01	28.75
Phr2	0.08	0.06	0.07	0.06	0.07	0.01	0.00	5.51
Pir1	0.04	0.05	0.05	0.05	0.05	0.00	0.00	4.92
Plb5	n.d.	0.01	n.d.	0.01	0.01	0.00	0.00	2.89
Rbt5	0.02	0.02	0.02	0.02	0.02	0.00	0.00	2.40
Rhd3	0.12	0.11	0.11	0.11	0.11	0.01	0.00	2.73
Sap9	0.03	0.05	0.05	0.01	0.04	0.02	0.01	26.81
Sod4	n.d.	0.02	n.d.	0.02	0.02	0.00	0.00	2.89
Ssr1	0.01	0.02	0.01	0.01	0.01	0.00	0.00	16.96
Utr2	0.03	0.05	0.05	0.02	0.04	0.02	0.01	19.30
Yps7	0.02	0.02	n.d.	0.01	0.02	0.01	0.00	17.00
Ywp1	0.04	0.02	0.02	0.05	0.03	0.02	0.01	23.88
						**median CV%**		**8.26**

median CV% **10.10**

Table S6. Detailed information on peptide identifications of the secretome.

			Glucose			Glucose + Lactate				Lactate			
**Protein**	**Residues**	**Peptides identified**	**R1**	**R2**	**R3**	**R1**	**R2**	**R3**	**R4**	**R1**	**R2**	**R3**	**R4**
Als fam	237-254	GLNDWNYPVSSESFSYTK	1	1	1								
Als1	176-188	STVDPSGYLYASR				1	1						
Asl4	77-91	FITDQTSIDLVADGR									1		
	255-284	TCTSTSVLVTYQNVPAGYRPFVDAYVSATR										1	
Bgl2	37-49	DVSTFEGDLDFLK	2	1									
	97-105	EALQNYLPK	2	2	1							1	
	106-112	ISVSTIK	2	2	1	2	1	1	1			1	1
	113-123	IFLVGSEALYR	1	1		2		1					
	124-134	EDLTASELASK	2		1		1		1		1	2	1
	261-288	AWGINVAVYEAFDEAWKPDTSGTSSVEK			1								
	289-297	HWGVWQSDK				2			1				
Cht1	148-162	DQTGYAALATQLR								2	1		
Cht2	92-98	TCQSLGK			2	2	2	2	2				
	99-121	TVLLSLGGGVGDYGFSDVASATK	2	2	1	2		2	1	1		1	
	122-129	FADTLWNK	1				1						
	229-236	FADSAPNK							1				
	240-258	LFVGVPATSNIAGYVDTSK	2	2	2		1	2	2	1	2		
	259-267	LSSAIEEIK											1
	268-290	CDSHFAGVSLWDASGAWLNTDEK					2						
Cht3	21-39	SNSNVAVYWGQNSGGSQQR	2	2	2	2	2	2	2	2	2	2	2
	100-121	TILLSLGGAAGSYSGFSDDATAK	2	2	1	2	2	2	2	2	1	2	2
	122-135	QFAHTLWDLFGNSK	1	2	2				2	1	2	2	2
	182-203	NYYLGAAPQCPYPDASVGPLLK				2	1	1	1	2	1	2	2
	246-255	LFVGVPASSR			1								
	256-279	AAGSGYNDPSAVSQYLTSDILNSK								2	1		
	280-307	YFGGISMWDVSAGWSNTNSNGNFVENMK				1							
Dag7	185-198 [185-199]	SVGNNPNHNPLCNK [SVGNNPNHNPLCNKK]	2	2	2	2	2	2	2	2	2	2	2
	208-216	SVDVTVVDR	2	2	1	2	2	2	2		2	2	2
	217-234	CEGCAYNDLDFSPAAFDK	2	2	2					2		2	1
	235-242	LADESLGR	1			1		1					
Ecm33	139-168	TGLTAGITSAESVVISDTGLSSLTGINVFK				1							
	171-202	VFNVNNNDNIDTIDSGLQEVTDTIDISYNAEK			1								
	246-262	VELAELTSIGNSLTINK		1									
	276-290	TIGGALQISDNSELR	2	2	1				1	2	2	2	2
	334-341	LSCSAFNK	1	1									
Eng1	430-442	AIDTNAPPTVFAR	2	1				2	2	1		1	
	507-516	VFGSINTNNK	1			1			1		2		1
	517-540	GVASYYFNPTNNAELIFSATSFSK										1	
	703-710	FSYTTAGK			2	1		2	1	2	1	2	1
	808-817	NMNSNYFSGK						1	1				
	914-923	LGGTWAQSNK	2	1	2	1		2	2				
	924-931	DWVNSLVR							1				

Fet3	256-263	YGVLITTIK	1										
Gca1	108 - 132	LNVHIEPTDLTDVFVLPEELVVKPK											1
	193-214	GHSITGLGESIHGSLNEPGVVK								2	2	2	
	270-286	ALSGVIDLYFFSGPDPK				1				1	1	2	
	314 - 330	WGYDTVESLETVVENFK										1	
	479 - 503	YFDNPVHPPFEVGYSGSDYPLGFDK									1		
	606-612	RPFIIGR										1	2
	817 - 834	GGNIIPTQEPGYTTTESR										1	
	835 - 853	KNPFGLLVALDAEGTASGK											1
Ilv5	171 - 183	TLYFSHGFSPVFK								1			
Lip4	161 - 171	QSGQAVLNSIR								1			
Mp65	128-141	GITYSPYSDNGGCK							1	2	2	1	2
	142-160	SESQIASEIAQLSGFDVIR	2	2	2	2	1	2	2	1	2	2	2
	161-174	LYGVDCDQVSAVLK	2	2	2	1	2	2	2	1	2	1	1
	234-240 [234-241]	AYVDEGR [AYVDEGRK]	1	2	2	2	2	2	2	2	2	2	2
	302-309 [302-310]	VWTACSGK	2	2	2	2	2	2	2	2	2	2	
	311-322 [311-333]	NVLITETGWPSK [NVLITETGWPSKGDSNGVAVPSK]	1	2	1		1	2	1			1	2
	334-344	SNQQAAISSIK	2	2	2	2	2	2	2	2	2	2	2
	345-362	SSCGASALLFTAFNDLWK		1		2	2		1				
	363-371 [363-378]	ADGPYNAEK [ADGPYNAEKYGIYSN] (C-term)			1	1		1	2	1	2	2	2
Msb2	1154-1177	SALNYPFVVENSISSAQIFQYLPR			1								
	1181-1187	YPFNGDK		1	1								
	1223-1238	ALGSFITTPGSAIYR	1	2	1		1						
	1239-1253	NPDSVLQALASLIDSR		2								1	
Nup	309-316	HGLVDYDR		1	1								
	383-400	NYVGDIFATLGGKPDFGK	2	2	2	2	1		1				
Op4	67 - 79	ENQIVTDVLTAIK									1		
	339-361	AATSGSSSGSSSNNADLNALINK								2			
orf19.1239	100-110	ISVDVIVGAIK									2		
	154-175	LTSLPNLIGNALGLNANTVSSK								2			
orf19.2460	239 - 256	AMIANNLIDLGNIVDAVK									1		
orf19.3499	28 - 42	TFGYPPPQYPEVTGK											1
orf19.4886	29-46	ALLLTPTSPVFTLLAIHK		1									
Pga4	99-116	AGIYVILDVNTPHSSITR			1								
Pga45	86-98	DANTEQTIEGILK				1	1	2	1				
	325-350	EIAALVGSLLSGDSSQTSQLSNYLGR				2	1	2	2				
Phr fam		SNTDASAFVK	2	2	1								1
Phr2	239-250	ASGYESATNDYK			1								

Pir1	249-266	ACSSANNLEMTLHDSVLK	1	1	1	2	2	2	1	2	2	2	2
	272-301	WGAIVANHQFQFDGPIPQAGTIYSAGWSIK		1	2	1				1	1	2	
Plb4.5	465-479	TNWPDGSALISSYER			1					1			
Pry1	328-337	FGENLAAGFK								1	1		
Rbe1	136-148	ALHGVPALSWSNK			1								
	222-229	STTQVGCAK			1					2	2	2	
	230-237	MMCSTAWR			1								
	236-347	QITVCEYLPR					1					1	
Rbt4	286-307	DGLSYSYGSSSVYNHFTQVVWK		1	2			1	1			1	
	348-358	SYMAENVLRPQ	1	1	2				1				
CFEM fam	47-56	IYDQLPECAK			1	1							
Rbt5	61-92	QSTSSTPCPYWDTGCLCVMPQFAGAVGNCVAK	1										
Rhd3	40-52	HEGAAIDYLFLGK					1			2	1	1	
	64-71 [59-71]	QIFQELK [YDDEKKQIFQELK]			1								
	73-78	TSSITVR	1	2	2	2		1		1	1		1
	79-103	QSFTLGGDVYELGATDNFIPVTINK				1		1					
	120-126	NVNDPYR			1								
	127-137	YSESEYAVSNK	1	1					1	2	1	2	
	140-151 [139-151]	TDDSAPITIVAK [KTDDSAPITIVAK]	1	2	1						1	1	1
Sap7	212-230	TGAFFLSLDNTQTLYTATLK								2	1	2	
	288 - 299	TDYSISYYDGDK											2
	375 - 390	DAVQGYILFGGIDHAK									1		
	458 - 474	DTPTLLDTGTTYSYLSK								1		2	
Scw4	401-418	QSSSGWNVVHTIAIGNER								1			
	419-437	VNAGAASVAQVQQAVDISK								2			
Scw11	282-298	SYGTDCGSLTTVLETCR	2	2	1	2	1	2	2	2	2	1	
	366-376	SQLQSAGYNGK		1	1	1	2	2	1				
	377-388	VTTSEPPATYLK	2	2	1	2	1	2	2	2	2	2	2
	417-436	DAGSYIVHQQQQVAEICGGK		1	2	2	2	2	2	2	2	2	2
	449-471	GSTLGVNVPSPENQEIAISSIIK		1	1	2			1				
	472-488	ETGGDCTILTTYNDFWK			1	1			1				
	489-506	DPGPYGIEQYFGVIQLFQ (C-term)			1				1				
Sim1	162-176	DGYYCSYACQAGMSK	2	2	2	2		2			1		
	177-196	TQWPSEQPSNGMSIGGLYCK				1	2	2	2	2			
	203-216	SNTDNDYLCEWGSK	2	2	2	2	2	2	2	2	2	2	2
	217-232	DVNFVSEISEDVAICR		2	1		2		1	1	2		1
	233-252	TDYPGSENMNIPTLLSAGGK	2	2	2	2	1	2	2	1			1
	253-265	APCSVVDGDTYFK	2	2	2								
	314-325	TYLSLIPNPNNK				1							
	326-333	DKPNYNNIK	2	2	1	2	2	2	2	1	1	2	1
	334-344	IVGDDVNGDCK		1	1		2	2	2	2	2	1	1

Ssr1	41-54	CSGLNDLSCICTTK	2	2	2	2	2	2	2	2	2	2	2
	64-79	EICPNGDADTAISAFK	1	2	2			1	2	1	2	1	2
Sun41	226-240	EGSYCSYACQSGMSK	2	2	2	2	2	2	2	2	2	2	2
	241-260	TQWPEDQPSNGVSIGGLLCK			2	2	2	2	2	2	2	2	2
	271-280 [271-281]	SNYLCEWGVKK	2	2	1	1	2	2	2	1			1
	282-287 [281-287]	KANVVNK		2	2	2	1						
	288-296	LSETVAICR	2	2	2	2	2	1	2	2	2	2	2
	297-331	TDYPGTENMVIPTVVGGGSTSVITVVDQSTYYTWR				2			1				
	390-395	DSLNFK	2	1			1						
	398-416	IVGESGSTVSGSCSYANGK		1		1		2	2				
Tos1	42-61	GIGFSGSYMDVTNMDENTGK	2	1	1	1				1	2	2	1
	62-85	CTQQLYSFSGNLSPLDEELSVHFR	2	2	1	1			1	2	1	2	1
	231-259	DSYYTPGSTDNCVFLNYHGGSGSGVWSAK	2	2	2	2	2	2	2	2	2	1	2
	260-287	FGNSLSYANADNSGGSSTPVPLEETTIK						1		1	1		1
	288-299	SGEEYIIFSGSK	1	1		1	1					1	2
	300-310	CGSSSDCGYYR	2	2	2	2	2	2	2	1	2	1	2
	311-320	GTVAYHGFK [KGTVAYHGFK]	2	1	2	2	2	2	2	2	2	2	2
	305-354	IFVFEFEMPSDTNGNGYNQDMPAVWLLNAK				1			2				
	358-370	TLQYGEATCSCWK		1		1		1	2	2	2	2	2
	371-388	TGCGELDLFEVLSSGSNK	2	1	2	2	2	2	2	1	1	2	2
	389-423	MISHLHDGQGSSQNSNNGGGGSQDYFERPTSGTFK	1		1		1				1	1	2
Utr2	245-253	YDYPQTPSR	1	2	2	2	2	2	2	2	2	2	2
	288-296	YGYYYAHIK							1				
	297-310	EIYATAYDIPNDVK	2	2	2	1	1		2	2	2	2	2
Xog1	39-54	GGGHNVAWDYDNNVIR			1	1	2	1	2	2	2	2	2
	108-119	HWSTWITEQDFK				1	2	2					
	120-130	QISNLGLNFVR			1	1		1				1	
	168-189	VWIDLHGAPGSQNGFDNSGLR					1	1					
	243-253	QFFLDGYNSLR			1	1	2		1				
	304-320	NINDHISVACNWGWDAK							1	2		2	2
	322-340	ESHWNVAGEWSAALTDCAK								1		1	
	341-347	WLNGVNR				2			2				
	351-378	YEGAYDNAPYIGSCQPLLDISQWSDEHK									1		
Ywp1	87-93	VINVPAR	1			1	2	2	2				
	94-110	NLYGAGAVPFFQVHLEK		2	2	1	1		2	1			
**Total peptide count**			**102**	**114**	**119**	**111**	**90**	**98**	**116**	**118**	**100**	**109**	**94**
contaminant peptides			2	1	2	1	0	0	0	11	0	3	3

Glucose

name	R1	R2	R3	average	SD	SE	CV%
Asl4	n.d.	n.d.	n.d.				
Bgl2	0.09	0.05	0.03	0.06	0.03	0.02	27.52
Cht1	n.d.	n.d.	n.d.				
Cht2	0.05	0.04	0.04	0.04	0.01	0.00	9.57
Cht3	0.05	0.05	0.05	0.05	0.00	0.00	2.07
Dag7	0.07	0.05	0.04	0.05	0.01	0.01	14.21
Ecm33	0.03	0.04	0.02	0.03	0.01	0.01	19.93
Eng1	0.05	0.02	0.03	0.03	0.02	0.01	27.21
Fet3	0.01	n.d.	n.d.	0.01			
Gca1	n.d.	n.d.	n.d.				
Ilv5	n.d.	n.d.	n.d.				
Lip4	n.d.	n.d.	n.d.				
Mp65	0.10	0.11	0.10	0.10	0.01	0.00	4.73
Msb2	0.01	0.04	0.03	0.03	0.02	0.01	37.45
Nup	0.02	0.03	0.03	0.02	0.00	0.00	8.76
Op4	n.d.	n.d.	n.d.				
orf19.4886	n.d.	0.01	n.d.	0.01			
orf19.1239	n.d.	n.d.	n.d.				
orf19.2460	n.d.	n.d.	n.d.				
orf19.3499	n.d.	n.d.	n.d.				
Pga4	n.d.	n.d.	0.01	0.01			
Pga45	n.d.	n.d.	n.d.				
Phr fam	0.02	0.02	0.01	0.02	0.01	0.00	22.67
Phr2	n.d.	n.d.	0.01	0.01			
Pir1	0.01	0.02	0.03	0.02	0.01	0.00	25.39
Plb4.5	n.d.	n.d.	0.01	0.01			
Pry1	n.d.	n.d.	n.d.				
Rbe1	n.d.	n.d.	0.03	0.03			
Rbt4	0.01	0.02	0.03	0.02	0.01	0.01	34.51
Rbt5	0.01	n.d.	n.d.	0.01			
Rhd3	0.03	0.04	0.04	0.04	0.01	0.00	11.81
Sap7	n.d.	n.d.	n.d.				
Scw4	n.d.	n.d.	n.d.				
Scw11	0.04	0.06	0.07	0.06	0.01	0.01	15.25
Sim1	0.10	0.11	0.09	0.10	0.01	0.01	6.38
Ssr1	0.03	0.04	0.03	0.03	0.00	0.00	5.20
Sun41	0.08	0.09	0.08	0.08	0.01	0.00	4.53
Tos1	0.14	0.10	0.09	0.11	0.02	0.01	13.16
Utr2	0.03	0.04	0.03	0.03	0.00	0.00	5.20
Xog1	n.d.	n.d.	0.03	0.03			
Ywp1	0.01	0.02	0.02	0.01	0.00	0.00	16.76
						**median CV%**	**13.69**

Glucose + Lactate

name	R1	R2	R3	R4	average	SD	SE	CV%
Asl4	0.01	0.01	n.d.	n.d.	0.01	0.00	0.00	6.93
Bgl2	0.05	0.02	0.02	0.03	0.03	0.02	0.01	25.70
Cht1	n.d.	n.d.	n.d.	n.d.				
Cht2	0.04	0.09	0.06	0.05	0.06	0.02	0.01	18.22
Cht3	0.06	0.05	0.05	0.06	0.06	0.01	0.00	4.64
Dag7	0.04	0.04	0.05	0.03	0.04	0.01	0.00	7.84
Ecm33	0.01	n.d.	n.d.	0.01	0.01	0.00	0.00	1.24
Eng1	0.03	n.d.	0.07	0.07	0.06	0.03	0.01	22.52
Fet3	n.d.	n.d.	n.d.	n.d.				
Gca1	n.d.	n.d.	n.d.	n.d.				
Ilv5	n.d.	n.d.	n.d.	n.d.				
Lip4	n.d.	n.d.	n.d.	n.d.				
Mp65	0.11	0.13	0.13	0.13	0.12	0.01	0.01	4.77
Msb2	n.d.	0.01	n.d.	n.d.	0.01			
Nup	0.02	0.01	n.d.	0.01	0.01	0.00	0.00	19.34
Op4	n.d.	n.d.	n.d.	n.d.				
orf19.4886	n.d.	n.d.	n.d.	n.d.				
orf19.1239	n.d.	n.d.	n.d.	n.d.				
orf19.2460	n.d.	n.d.	n.d.	n.d.				
orf19.3499	n.d.	n.d.	n.d.	n.d.				
Pga4	n.d.	n.d.	n.d.	n.d.				
Pga45	0.03	0.02	0.04	0.03	0.03	0.01	0.00	14.42
Phr fam	n.d.	n.d.	n.d.	n.d.				
Phr2	n.d.	n.d.	n.d.	n.d.				
Pir1	0.03	0.02	0.02	0.01	0.02	0.01	0.00	19.82
Plb4.5	n.d.	n.d.	n.d.	n.d.				
Pry1	n.d.	n.d.	n.d.	n.d.				
Rbe1	n.d.	0.01	n.d.	n.d.	0.01			
Rbt4	n.d.	n.d.	0.01	0.02	0.01	0.00	0.00	18.13
Rbt5	n.d.	n.d.	n.d.	n.d.				
Rhd3	0.03	0.01	0.02	0.01	0.02	0.01	0.00	25.37
Sap7	n.d.	n.d.	n.d.	n.d.				
Scw4	n.d.	n.d.	n.d.	n.d.				
Scw11	0.10	0.07	0.08	0.09	0.08	0.01	0.01	8.25
Sim1	0.09	0.12	0.12	0.09	0.11	0.02	0.01	7.94
Ssr1	0.02	0.02	0.03	0.03	0.03	0.01	0.00	14.70
Sun41	0.11	0.11	0.09	0.09	0.10	0.01	0.00	4.24
Tos1	0.12	0.11	0.10	0.11	0.11	0.01	0.00	2.71
Utr2	0.03	0.03	0.02	0.04	0.03	0.01	0.00	15.69
Xog1	0.05	0.08	0.05	0.05	0.06	0.01	0.01	10.36
Ywp1	0.02	0.03	0.02	0.03	0.03	0.01	0.00	15.99
							**median CV%**	**14.42**

Lactate

name	R1	R2	R3	R4	average	SD	SE	CV%
Asl4	n.d.	0.01	0.01	n.d.	0.01	0.00	0.00	3.04
Bgl2	n.d.	0.01	0.04	0.02	0.02	0.01	0.01	29.57
Cht1	0.02	0.01	n.d.	n.d.	0.01	0.00	0.00	18.23
Cht2	0.02	0.02	0.01	0.01	0.01	0.01	0.00	18.10
Cht3	0.08	0.07	0.07	0.09	0.08	0.01	0.00	4.25
Dag7	0.03	0.04	0.06	0.05	0.05	0.01	0.01	11.25
Ecm33	0.02	0.02	0.02	0.02	0.02	0.00	0.00	4.94
Eng1	0.03	0.03	0.04	0.02	0.03	0.01	0.00	11.65
Fet3	n.d.	n.d.	n.d.	n.d.				
Gca1	0.03	0.04	0.06	0.04	0.04	0.02	0.01	18.59
Ilv5	0.01	n.d.	n.d.	n.d.	0.01			
Lip4	0.01	n.d.	n.d.	n.d.	0.01			
Mp65	0.09	0.14	0.12	0.14	0.12	0.02	0.01	8.88
Msb2	n.d.	n.d.	0.01	n.d.	0.01			
Nup	n.d.	n.d.	n.d.	n.d.				
Op4	0.02	0.01	n.d.	n.d.	0.01	0.00	0.00	18.23
orf19.4886	n.d.	n.d.	n.d.	n.d.				
orf19.1239	0.02	0.02	n.d.	n.d.	0.02	0.00	0.00	5.84
orf19.2460	n.d.	0.01	n.d.	n.d.	0.01			
orf19.3499	n.d.	n.d.	n.d.	0.01	0.01			
Pga4	n.d.	n.d.	n.d.	n.d.				
Pga45	n.d.	n.d.	n.d.	n.d.				
Phr fam	n.d.	n.d.	n.d.	0.01	0.01			
Phr2	n.d.	n.d.	n.d.	n.d.				
Pir1	0.03	0.03	0.04	0.02	0.03	0.01	0.00	11.65
Plb4.5	0.01	n.d.	n.d.	n.d.	0.01			
Pry1	0.01	0.01	n.d.	n.d.	0.01	0.00	0.00	5.84
Rbe1	0.02	0.02	0.03	n.d.	0.02	0.01	0.00	12.66
Rbt4	n.d.	n.d.	0.01	n.d.	0.01			
Rbt5	n.d.	n.d.	n.d.	n.d.				
Rhd3	0.04	0.04	0.04	0.02	0.04	0.01	0.00	13.53
Sap7	0.03	0.02	0.04	0.02	0.03	0.01	0.00	14.69
Scw4	0.03	n.d.	n.d.	n.d.	0.03			
Scw11	0.05	0.06	0.05	0.04	0.05	0.01	0.00	7.62
Sim1	0.08	0.08	0.05	0.06	0.07	0.02	0.01	11.57
Ssr1	0.03	0.04	0.03	0.04	0.03	0.01	0.00	12.77
Sun41	0.06	0.06	0.06	0.07	0.06	0.01	0.00	6.80
Tos1	0.10	0.14	0.13	0.18	0.14	0.03	0.02	11.95
Utr2	0.03	0.04	0.04	0.04	0.04	0.00	0.00	4.94
Xog1	0.04	0.03	0.06	0.04	0.04	0.01	0.01	12.03
Ywp1	0.01	n.d.	n.d.	n.d.	0.01			
							**median CV%**	**11.65**

median CV% **13.25**

References

Albrecht, A., Felk, A., Pichova, I., Naglik, J.R., Schaller, M., de Groot, P., Maccallum, D., Odds, F.C., Schafer, W., Klis, F., Monod, M. & Hube, B. 2006, "Glycosylphosphatidylinositol-anchored proteases of Candida albicans target proteins necessary for both cellular processes and host-pathogen interactions", *The Journal of biological chemistry,* vol. 281, no. 2, pp. 688-694.

Braun, B.R., Head, W.S., Wang, M.X. & Johnson, A.D. 2000, "Identification and characterization of TUP1-regulated genes in Candida albicans", *Genetics,* vol. 156, no. 1, pp. 31-44.

Davis, D., Edwards, J.E.,Jr, Mitchell, A.P. & Ibrahim, A.S. 2000, "Candida albicans RIM101 pH response pathway is required for host-pathogen interactions", *Infection and immunity,* vol. 68, no. 10, pp. 5953-5959.

Fonzi, W.A. & Irwin, M.Y. 1993, "Isogenic strain construction and gene mapping in Candida albicans", *Genetics,* vol. 134, no. 3, pp. 717-728.

Fu, Y., Ibrahim, A.S., Sheppard, D.C., Chen, Y.C., French, S.W., Cutler, J.E., Filler, S.G. & Edwards, J.E.,Jr 2002, "Candida albicans Als1p: an adhesin that is a downstream effector of the EFG1 filamentation pathway", *Molecular microbiology,* vol. 44, no. 1, pp. 61-72.

Garcera, A., Castillo, L., Martinez, A.I., Elorza, M.V., Valentin, E. & Sentandreu, R. 2005, "Anchorage of Candida albicans Ssr1 to the cell wall, and transcript profiling of the null mutant", *Research in microbiology,* vol. 156, no. 9, pp. 911-920.

Muhlschlegel, F.A. & Fonzi, W.A. 1997, "PHR2 of Candida albicans encodes a functional homolog of the pH-regulated gene PHR1 with an inverted pattern of pH-dependent expression", *Molecular and cellular biology,* vol. 17, no. 10, pp. 5960-5967.

Murad, A.M., Lee, P.R., Broadbent, I.D., Barelle, C.J. & Brown, A.J. 2000, "CIp10, an efficient and convenient integrating vector for Candida albicans", *Yeast (Chichester, England),* vol. 16, no. 4, pp. 325-327.

Negredo, A., Monteoliva, L., Gil, C., Pla, J. & Nombela, C. 1997, "Cloning, analysis and one-step disruption of the ARG5,6 gene of Candida albicans", *Microbiology (Reading, England),* vol. 143 (Pt 2), no. Pt 2, pp. 297-302.

Noble, S.M., French, S., Kohn, L.A., Chen, V. & Johnson, A.D. 2010, "Systematic screens of a Candida albicans homozygous deletion library decouple morphogenetic switching and pathogenicity", *Nature genetics,* vol. 42, no. 7, pp. 590-598.

Noble, S.M. & Johnson, A.D. 2005, "Strains and strategies for large-scale gene deletion studies of the diploid human fungal pathogen Candida albicans", *Eukaryotic cell,* vol. 4, no. 2, pp. 298-309.

Pardini, G., De Groot, P.W., Coste, A.T., Karababa, M., Klis, F.M., de Koster, C.G. & Sanglard, D. 2006, "The CRH family coding for cell wall glycosylphosphatidylinositol proteins with a predicted transglycosidase domain affects cell wall organization and virulence of Candida albicans", *The Journal of biological chemistry,* vol. 281, no. 52, pp. 40399-40411.

Plaine, A., Walker, L., Da Costa, G., Mora-Montes, H.M., McKinnon, A., Gow, N.A., Gaillardin, C., Munro, C.A. & Richard, M.L. 2008, "Functional analysis of Candida

albicans GPI-anchored proteins: roles in cell wall integrity and caspofungin sensitivity", *Fungal genetics and biology : FG & B,* vol. 45, no. 10, pp. 1404-1414.

Saporito-Irwin, S.M., Birse, C.E., Sypherd, P.S. & Fonzi, W.A. 1995, "PHR1, a pH-regulated gene of Candida albicans, is required for morphogenesis", *Molecular and cellular biology,* vol. 15, no. 2, pp. 601-613.

Selvaggini, S., Munro, C.A., Paschoud, S., Sanglard, D. & Gow, N.A. 2004, "Independent regulation of chitin synthase and chitinase activity in Candida albicans and Saccharomyces cerevisiae", *Microbiology (Reading, England),* vol. 150, no. Pt 4, pp. 921-928.

Smith, D.A., Nicholls, S., Morgan, B.A., Brown, A.J. & Quinn, J. 2004, "A conserved stress-activated protein kinase regulates a core stress response in the human pathogen Candida albicans", *Molecular biology of the cell,* vol. 15, no. 9, pp. 4179-4190.

Wilson, R.B., Davis, D. & Mitchell, A.P. 1999, "Rapid hypothesis testing with Candida albicans through gene disruption with short homology regions", *Journal of Bacteriology,* vol. 181, no. 6, pp. 1868-1874.
